# In-Vivo Quantitative Proteomics Reveals a Key Contribution of Post-Transcriptional Mechanisms to the Circadian Regulation of Liver Metabolism

**DOI:** 10.1371/journal.pgen.1004047

**Published:** 2014-01-02

**Authors:** Maria S. Robles, Jürgen Cox, Matthias Mann

**Affiliations:** Department of Proteomics and Signal Transduction, Max-Planck Institute of Biochemistry, Martinsried, Germany; Charité - Universitätsmedizin Berlin, Germany

## Abstract

Circadian clocks are endogenous oscillators that drive the rhythmic expression of a broad array of genes, orchestrating metabolism and physiology. Recent evidence indicates that post-transcriptional and post-translational mechanisms play essential roles in modulating temporal gene expression for proper circadian function, particularly for the molecular mechanism of the clock. Due to technical limitations in large-scale, quantitative protein measurements, it remains unresolved to what extent the circadian clock regulates metabolism by driving rhythms of protein abundance. Therefore, we aimed to identify global circadian oscillations of the proteome in the mouse liver by applying in vivo SILAC mouse technology in combination with state of the art mass spectrometry. Among the 3000 proteins accurately quantified across two consecutive cycles, 6% showed circadian oscillations with a defined phase of expression. Interestingly, daily rhythms of one fifth of the liver proteins were not accompanied by changes at the transcript level. The oscillations of almost half of the cycling proteome were delayed by more than six hours with respect to the corresponding, rhythmic mRNA. Strikingly we observed that the length of the time lag between mRNA and protein cycles varies across the day. Our analysis revealed a high temporal coordination in the abundance of proteins involved in the same metabolic process, such as xenobiotic detoxification. Apart from liver specific metabolic pathways, we identified many other essential cellular processes in which protein levels are under circadian control, for instance vesicle trafficking and protein folding. Our large-scale proteomic analysis reveals thus that circadian post-transcriptional and post-translational mechanisms play a key role in the temporal orchestration of liver metabolism and physiology.

## Author Summary

The circadian clock is an evolutionary system that allows organisms to anticipate and thus adapt to daily changes in the environment. In mammals, the circadian clock is found in virtually every tissue regulating rhythms of metabolism and physiology. While a lot of studies have focused in how circadian clocks regulate gene expression little is known about daily control of protein abundance. Here we applied state of the art mass spectrometry in combination with quantitative proteomics to investigate global circadian oscillations of the proteome in the mouse liver. We found that approximately 6% of the liver proteins are cycling daily and interestingly the majority of these oscillations diverge from the behavior of their transcripts. Our data indicates that post-transcriptional mechanisms play an essential role in shaping the phase of rhythmic proteins downstream of transcription regulation to ultimately drive rhythms of metabolism. Moreover, the contribution of post-transcriptional regulation seems to differ among distinct metabolic pathways. Overall we not only found circadian oscillations in the abundance of proteins involved in liver specific metabolic pathways but also in essential cellular processes.

## Introduction

Circadian clocks are endogenous self-sustained oscillators that drive daily rhythms of metabolism and physiology [1], [2]. In mammals the molecular mechanism underlying circadian oscillations is based on interconnected transcriptional and translational feedback loops that ultimately regulate the rhythmic expression of clock controlled genes [2]. Gene expression studies in central (suprachiasmatic nucleus of the hypothalamus) and peripheral tissues have revealed thousands of rhythmic transcripts that are associated with daily control of metabolism [3]–[7]. In particular, the hepatic clock drives transcriptional oscillations of genes that are essential for local metabolism regulating glucose, cholesterol and bile acids homestostasis [8], [9]. In this regard, daily rhythms of metabolites have been recently described in the mouse liver [10], [11]. In human plasma and saliva metabolite cycles are reported to be independent of sleep and food intake [12]. In contrast to these investigations of the transcriptome and the metabolome, circadian protein oscillations have not been accessed at a large scale mainly due to the technological limitations in the measurement of protein abundance in a high-throughput and accurate manner. For instance, a protein expression study of mouse liver using two-dimensional (2D) gel electrophoresis at four circadian time points detected 60 rhythmic spots, of which 39 could be identified as protein products [13]. Because recent evidence suggests that circadian metabolism is also influenced by post-transcriptional mechanisms [14]–[18], it would be desirable to study the circadian dynamics of the proteome at a large scale.

Mass spectrometry (MS)-based proteomics [19] has developed rapidly in recent years and its quantitative accuracy has improved dramatically [20]. It is increasingly applied not only to cell lines but also to more complex systems such as tissues, where accurate quantification with technologies like Stable Isotope Labeling by Amino acids in Cell culture (SILAC) has now become possible [21], [22]. Here we aimed to identify global daily changes in protein abundance in the mouse liver by applying high resolution MS-based proteomics in combination with quantification via the in vivo SILAC mouse technology [23]. Mixing pooled livers from fully ‘heavy’ labeled SILAC mice with liver samples collected over two 24 h cycles enabled us to accurately quantify the abundance of thousands of proteins. Metabolic processes were particularly well covered and turned out to be under extensive circadian control by means of protein abundance. Bioinformatic analysis highlighted significant divergence between the circadian transcriptome and proteome, including protein abundance changes without corresponding message changes and large differences in the phase of abundance. We also focus on particular biological processes that seem to be tightly regulated at the post-transcriptional level and that may have practical implications such as rhythms of detoxifying enzymes that are relevant to chronotherapy.

## Results

### High Throughput SILAC-Based Quantitative Proteomics of the Mouse Liver

**Figure 1:**
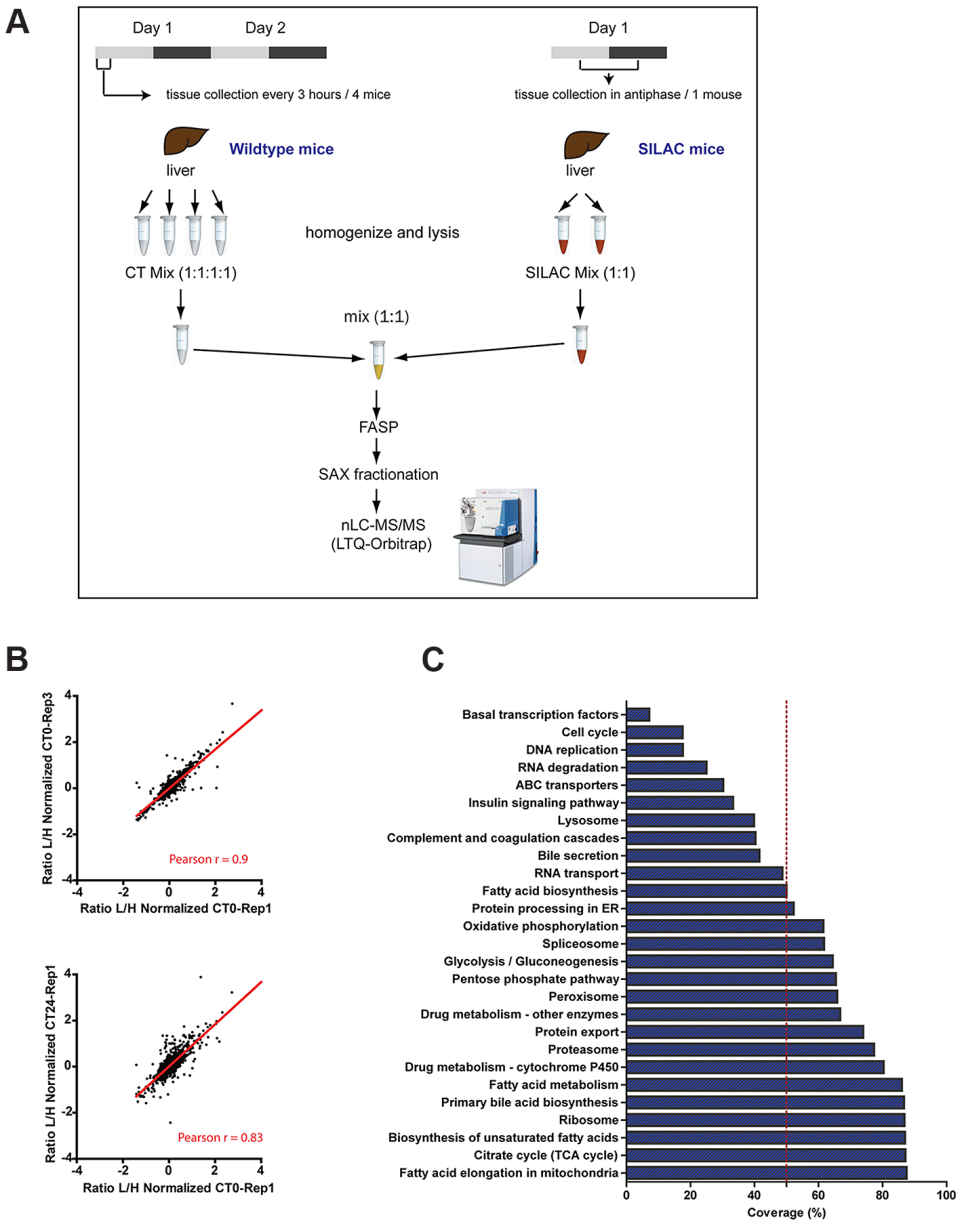
SILAC-based circadian proteome of the mouse liver. (A) Schematic representation of the workflow followed to perform the quantitative proteomics analysis of mouse livers harvested sixteen times across two circadian cycles. (B) Sample replicates show high degree of correlation. Scatter plots showing logarithmic normalized protein ratios (Light/High SILAC) in replicate measurements, technical (upper) and biological (lower). Red line shows linear regression of the data and the calculated correlation coefficient (Pearson r) for each pair of replicates is indicated at the bottom. (C) The quantified proteome contains proteins from a broad range of metabolic and cellular processes. Graph shows the percentage of protein coverage for different KEGG pathways observed in the quantified dataset proteome used for the circadian analysis.

Liver samples were harvested from wildtype C57BL/6 mice kept one day in constant darkness after being entrained to a 12–12 h light-dark schedule. Protein lysates were prepared from the mouse livers, which were collected at intervals of 3 h over two circadian cycles (4 mice per time point; n = 64 total mice). For each time point, protein extracts from the four livers were mixed in equal amounts to have a single sample per time point. We decided to use SILAC in an in vivo format for the proteomic quantification method [23], [24]. An internal, spike-in standard mix was constructed by combining equal amounts of protein lysates from liver samples of two heavy SILAC labeled mice collected in anti-phase (see Material and Methods). The pooled lysates of each of the 16 time points was mixed with equal amounts of the internal standard prior to digestion. Resulting peptides were then separated into six fractions and measured on a linear ion trap – Orbitrap mass spectrometer (Figure 1A). The experiment was performed in technical triplicates resulting in 288 liquid chromatography (LC)-MS/MS files that were subsequently processed in the MaxQuant software environment [25]. The relative abundance of the liver proteome is calculated for each time point by taking the ratio of the mass spectrometric signal for individual proteins to the signal of the spiked in heavy SILAC standard. For circadian analysis we filtered out those protein groups with accurate quantification values in less than half of the samples measured; the resulting dataset contained 3132 protein groups (Table S1).

Reproducibility was tested by comparing the measurements to each other and calculated Pearson correlation coefficients. For all of the comparisons we obtained high r values (between 0.6 and 0.93) as illustrated for technical and biological replicates in Figure 1B. The stringently quantified proteome dataset of 3132 contains proteins from a broad range of metabolic and cellular processes. Coverage of transcription factors and cell cycle proteins in these post-mitotic cells was relatively low, as expected. We did not obtain quantification values for clock proteins, likely due to their low abundance particularly at some times of the cycle. Therefore proper entrainment of the mice was confirmed by assessing the expression profile of *Bmal1* and *Per2* mRNA as well as of PER2 protein in the collected liver samples (Figure S1). Nevertheless, the quantified liver proteome covered extensively many liver specific pathways like fatty acid and drug metabolism (Figure 1C). In addition, more than 50% of coverage was observed for more general cellular processes and components such the ribosome, proteasome and spliceosome (Figure 1C).

### Circadian Oscillations of the Mouse Liver Proteome

To identify proteins with circadian rhythms of abundance in the mouse liver quantified dataset we adapted a statistical algorithm (Materials and Methods). Specifically, we determined goodness-of-fit of the expression ratios to a cosine curve with a period of 23.6 hours, the circadian period reported for the used mouse strain [26]. To calculate the rate of false discovery the experimental data were repeatedly scrambled and fitted to the cosine curve, preserving the technical triplicates in the randomization. This allows an estimate of the frequency that a false observation matches the curve by chance. With this statistical analysis, at a false discovery rate (FDR or q-value) <0.33, we identified 186 proteins (Table S2) from the total 3132 dataset (Table S1) that showed circadian rhythmic profiles of abundance in the mouse liver across two consecutive cycles. Based on this analysis, we thus estimate that at least 6% of our mouse liver proteome dataset shows circadian oscillations. We additionally compared the statistical analysis performed by our method to the JTK Cycle method, a standard method used in the circadian field to detect rhythmicity [27]. The results indicated an excellent correlation (Pearson r>0.9) between the two methods for q-value estimation in the total quantified dataset (Figure S1C) as well as phase determination of statistical significant cycling proteins (Figure S1D). This shows that the statistical algorithm to detect oscillations included in the Perseus software package may be widely applicable in circadian studies.

**Figure 2:**
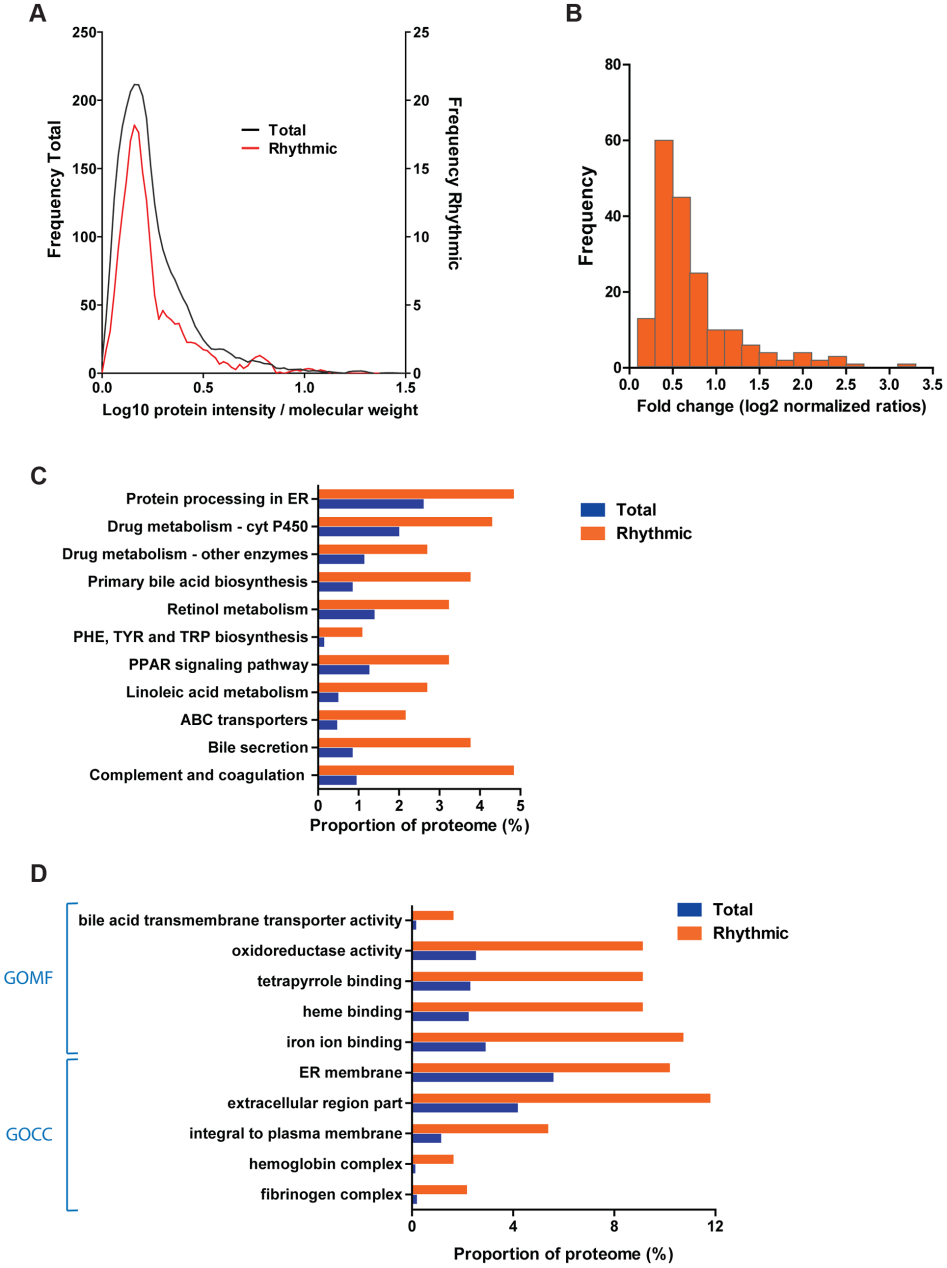
The liver circadian proteome. (A) Liver cycling proteins are not biased towards high abundant proteins. Graph shows the protein abundance distribution of the quantified (black line) and rhythmic (red line) proteome of the liver calculated based on the protein intensity and molecular weight. (B) The majority of cycling proteins change in abundance less than two fold across the cycle. Histogram of protein fold change for the cycling liver proteome calculated based on the expression ratios obtained for each time point measured. (C) Several liver specific metabolic pathways are enriched among the liver cycling proteome. Histogram shows the proportion of proteins from the total quantified (blue) and rhythmic (orange) proteome annotated in the indicated selected KEGG pathways. All the specified categories are statistically enriched in the cycling proteome (Fisher exact test *p*<0.05) (D) Overrepresentation of proteins associated to cellular insoluble and extracellular fractions in the circadian liver proteome. The graph shows the proportion of proteins annotated to the indicated selected Gene Ontology categories (left), statistically enriched (Benjamini Hochberg FDR<0.05) in the rhythmic (orange) compared to the total quantified (blue) proteome.

We assessed the total abundance of each protein in the samples by using the added peptide intensity obtained in the MS analysis normalized to their molecular weight. The abundance profile of the cycling proteome was very similar to the one observed for the entire quantified proteome (Figure 2A). This indicates that cycling proteins identified in our analysis are not biased against low abundance. Although the amplitude of oscillations has been studied extensively at the transcript level, there is currently no information about the accurate fold change of the cycling proteome. Using the logarithmic normalized expression ratios obtained for every data point, we calculated the amplitude of the abundance for each oscillating protein across the circadian day. The mean of the distribution of the total fold change for rhythmic proteins in the mouse liver is 1.38; therefore the large majority of rhythmic proteins change less than two fold (Figure 2B). The abundance of cycling proteins does not correlate with the coefficient of variation of the oscillation (Pearson *r* = 0.018), indicating that circadian rhythms were equally detectable in high and low abundance proteins. Next we tested whether there were categorical protein annotations significantly different from the overall fold change distribution by applying a recently developed annotation enrichment algorithm [28]. Proteins cycling with small amplitude are statistically enriched in those annotated as being modified by acetylation as well as ubiquitin-like modifier proteins whereas glycosylated proteins show cycles with large amplitudes (Benjamini Hochberg FDR<0.05 for all; Figure S1E).

Enrichment analysis of the circadian proteome compared to the total quantified proteome revealed that several essential liver metabolic Kyoto Encyclopedia of Genes and Genomes (KEGG) pathways such as drug and bile acids metabolism were over-represented (Fisher test p<0.05) (Figure 2C, Table S3). Interestingly, a significant fraction of the membrane bound proteome (Figure 2D, Table S3) is under circadian regulation in the mouse liver. In addition, secreted proteins from both extracellular matrix as well as plasma- as those involved in blood coagulation- all synthesized in the liver, are also enriched (Fisher test p<0.01) in the circadian proteome suggesting that the circadian clock in the liver may play a role in the generation of rhythms of plasma and extracellular proteins (Figure 2C and 2D, Table S3).

Taken together, we report here the most comprehensive and accurately measured circadian proteome of the mouse liver to date and find that it is significantly enriched in essential protein categories whose temporal regulation has not been previously documented.

### Phase Analysis of Circadian Control of Liver Protein Abundance

**Figure 3:**
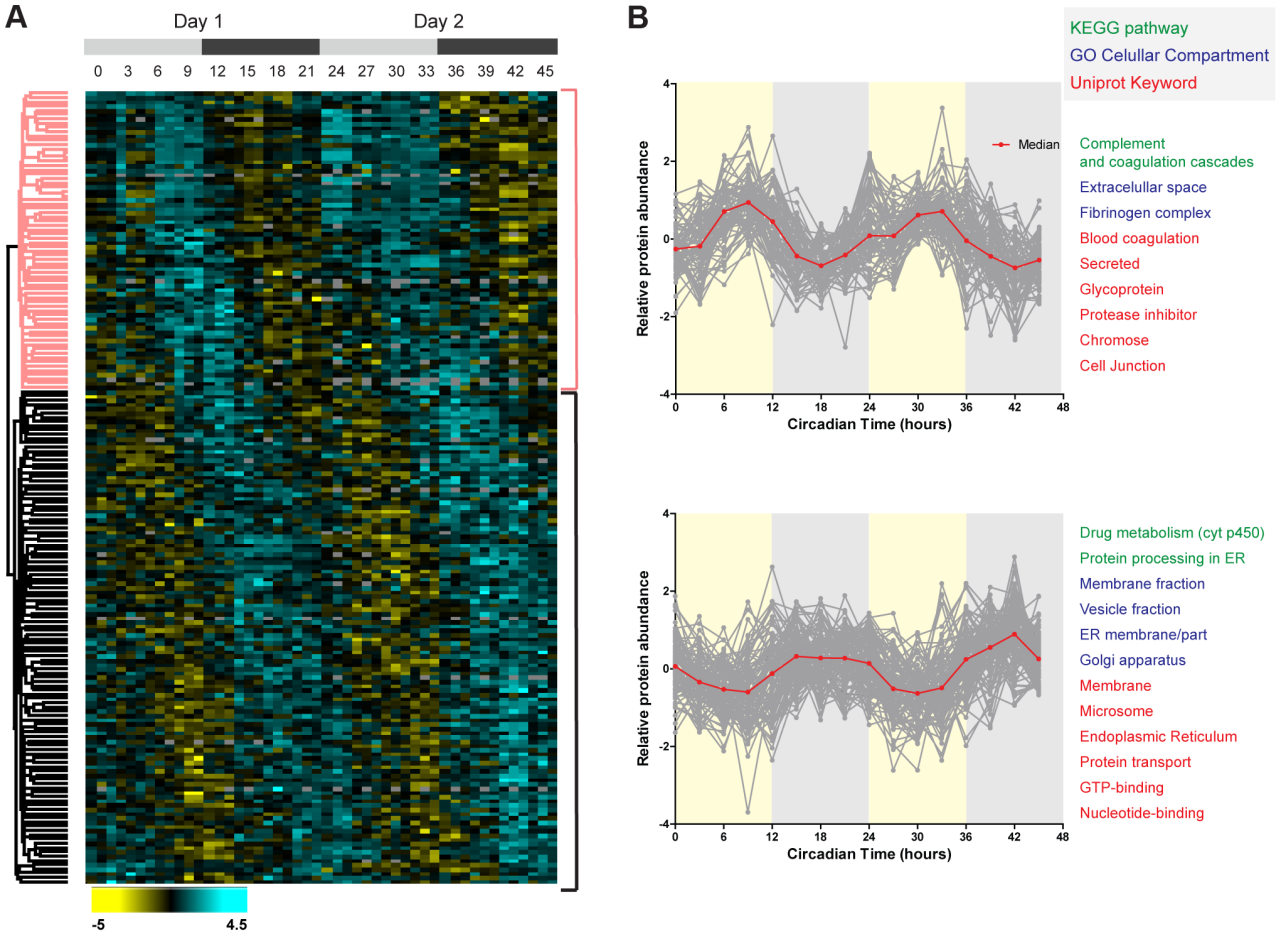
Temporal profile of the mouse liver proteome across two consecutive cycles. (A) Hierarchical clustering of daily rhythmic proteins in mouse liver according to the phase of maximal expression. Values for each protein (rows) at all the circadian times analyzed (columns) are colored based on the abundance ratios, high (light blue) and low (yellow) values (Z-scored normalized ratios) are indicated in the color scale bar at the bottom. Top gray bars indicate the circadian day (light gray) and night (dark gray) of the two consecutive sampled cycles. (B) Plots display the abundance profile of proteins (grey lines) from the day (upper) and night (lower) branches of the dendrogram across the two circadian cycles. Red line represented the calculated median profile in each cluster. Protein categories enriched (FDR<0.05) in each protein cluster are indicated on the right side using a color to indicate the annotation database they belong to.

We performed phase dependent hierarchical cluster analysis of the mouse liver circadian proteome using the normalized logarithmic expression ratios for all sampled circadian times (CT). The heat map representation of the ratios showed cycling proteins with phases distributed across the two consecutive days (Figure 3A). The overall pattern of these phases was similar to previously reported circadian profiles of transcripts in this organ [3]–[6]. The analysis resulted in two major branches in the dendrogram which mostly segregate proteins peaking during the day or during the night (Figure 3A, 3B). Almost 2/3 of the cycling proteome displayed night phases (CT12 to CT24) and 1/3 day phases (CT0 to CT12). Mice are nocturnal animals hence behavioral and metabolic activity is predominant during the dark phase, which may explain the larger number of night peaking proteins in the liver. We next asked if any protein category was statistically different in these two main clusters, using a Fisher exact test cut-off of p<0.02. Secreted and extracellular proteins tended to be rhythmic with abundance peak during the day while proteins associated to membrane, to the endoplasmic reticulum (ER) as well as to the Golgi apparatus mainly peaked at night. Moreover, proteins from complement and coagulation cascades oscillated with exclusively day phases (Figure 3B and S2A) while many rhythmic proteins with nocturnal phases are involved in drug metabolism, bile secretion and protein processing in ER (Figure 3B and S2B).

To identify additional phase dependent enriched categories of cycling proteins in the mouse liver we directly tested for phase enrichment of categorical annotations in a wider set (838) of cycling candidates using a loser q-value cut-off (<0.66). To determine the significance of the cycling annotation distribution test, we employed a Fisher Exact Test cut-off of FDR<0.02, resulting only in categories of proteins with phases highly significant clustered at specific time of the circadian cycle. Day-enriched Gene Ontology Cellular Component (GOCC) protein categories comprised mainly nuclear and extracellular proteins while mitochondrial associated proteins peaked at the day-night interphase (Figure S2C). In contrast, proteins associated to Golgi displayed statistically enriched night phases. The cycling annotation distribution analysis also highlighted KEGG metabolic pathways enriched at specific times of the circadian cycle. Drug metabolism and protein processing in ER were remarkably enriched with nocturnal phases which agree with the exclusive presence of proteins from these pathways in the night hierarchical cluster described above (Figure 3A, S2B and S2D). The complement and coagulation cascades showed, in contrast, day-enriched phases as mentioned above too (Figure 3A, S2A and S2D).

Together our enrichment analyses revealed that a broad range of cellular and metabolic components are subjected to temporal regulation by means of protein abundance. A considerable number of cycling proteins belong to categories that have not previously been described to be under circadian control in the mouse liver.

### Divergence between the Liver Circadian Transcriptome and Proteome

**Figure 4:**
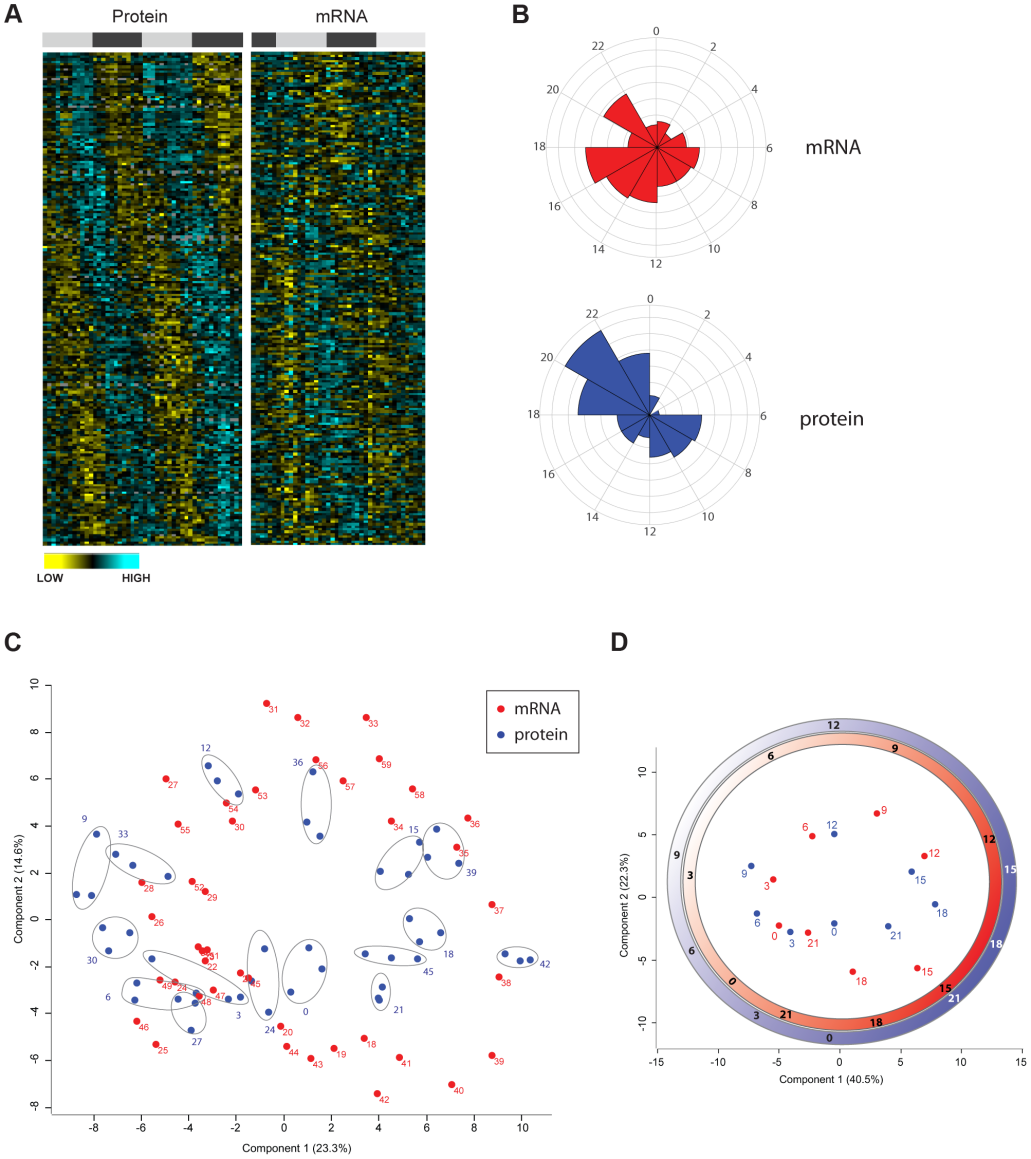
Divergence in the temporal profile of mouse liver proteome and transcriptome. (A) Heat maps of circadian rhythmic proteins ordered by the phase of maximal expression as in Figure 3A (left panel) and values for their corresponding transcripts (right panel) obtained from the data published by Hughes et al. [3]. (B) Frequency distribution of abundance phases for mouse liver rhythmic mRNAs (top panel) and their corresponding cycling proteins (bottom panel) across the circadian day. (C) Principal component analysis of the circadian proteome (blue) as well the transcriptome (red) based on their abundance values. Data of technical triplicate measurements of the proteome for each time point are grouped with grey ellipses. (D) Graphical representation of the time coordinates of a 24 hour cycle (as in an analog clock) calculated based on the proteome (red) and transcriptome (blue) data. Time delay between transcriptome and proteome can be observed with strikingly different intervals across the circadian cycle.

Circadian rhythms of mRNA in the mouse liver have been widely studied, however it still remains unresolved whether and how the temporal changes in transcripts translate to global oscillations of protein abundance. To this end, we compared our circadian proteome to a microarray study of the mouse liver transcriptome from the Hogenesch group [3]. That experiment was performed with 1 h resolution and around 10% of the transcriptome was found to be cycling, depending on the statistical algorithm employed. We matched each protein group of our quantified dataset to its corresponding Affymetrix entries which gave us a final dataset of 3046 protein groups. Using the same statistical algorithm that we applied to the proteome we assessed both circadian oscillations for proteins and transcripts. By using a cut-off of q-value<0.33 we identified 181 protein groups (6%) with significant circadian rhythmicity, among them 151 also showed rhythms at the mRNA level (Figure 4A, Table S4). Comparison of q-value distributions for the total dataset of protein and mRNA indicates that most of the cycling proteins with arrhythmic mRNA showed q-values for their transcripts far from the applied cut-off (Figure S3A). Thus, around 20% of cycling proteins do not seem to be accompanied by statistical significant oscillations of their mRNA based on the gene array data. Circadian gene expression can also be assessed by analyzing cycling binding patterns of CLOCK and BMAL transcription factors in promoters. Therefore, in addition to this gene expression study we used a recently reported set of BMAL1 target genes found by chromatin immunoprecipitation (ChIP) [29]. In that data set, we found only four additional genes where the corresponding protein was cycling but the transcript did not show statistically significant oscillation. Together our statistical analyses suggest that around 20% of the cycling proteins do not show circadian regulation of their corresponding transcript, implying wide-spread circadian post-transcriptional control of protein abundance.

Given that very recent evidence indicated that post-transcriptional mechanisms appear to largely determine the phase of mRNA oscillations [16], [30]–[32], we globally explored the distribution of phases for oscillating transcripts and proteins. We found that phases of rhythmic transcripts matching cycling proteins in the mouse liver were evenly distributed throughout the cycle with higher frequency during the night (Figure 4B upper graph) similarly to what was previously reported for the total circadian liver transcriptome [3]–[6]. In contrast, the phases of cycling proteins with rhythmic transcripts were distributed in two main clusters, a smaller one centered at the middle of the day and a larger one in the second part of the night. Surprisingly, very few proteins peaked in the first hours of the day (Figure 4B lower graph). The divergence in the distribution of transcript and protein phase suggests that the cycles of protein abundance in the mouse liver do not necessarily reflect mRNA changes and instead are influenced by post-transcriptional mechanisms. For the clock genes themselves, a characteristic time delay (usually 4–6 h) between mRNA and protein expression has been described [17]. However, it is poorly understood whether this is a general feature of all cycling genes and proteins. To address this question using the quantitative nature of our circadian proteome data, we calculated the time delay between the peak of expression of each rhythmic transcript and its oscillating protein. The total distribution of time delays showed with a preferential window of 2 to 6 h between transcript and protein peaks for almost 50% of the cycling mRNA and proteins (Figure S3C). Strikingly, around 40% of oscillating proteins peaked more than 6 h later than their corresponding transcripts. This data indicates that regulation of time delay between peaks of mRNA and protein is an important feature of circadian biology, implying general post-transcriptional mechanisms that define the overall phase-tuning of the proteome.

Principal component analysis (PCA) of the cycling proteome and transcriptome showed excellent clustering of the technical triplicates and biological replicates in the two-dimensional graph (Figure 4C). The graphical representation of the data on the basis of the two main PCA components resembles an analog clock (Figure 4D), indicating that time is the main component accounting for the overall difference between both datasets. We then calculated the angles corresponding to the median value for each time point experimentally analyzed in the proteome and for the published transcriptome. These angles directly visualize (Figure 4D) the characteristic time delay between mRNA and protein cycles mentioned above (Figure S3C). Very interestingly, the length of this time lag varies across the circadian cycle (Figure 4D). Together the comparison of the phases of the transcriptome vs. the proteome clearly demonstrates that circadian protein oscillations are shaped post-transcriptionally.

### Different Contribution of Circadian Post-transcriptional Control among Diverse Metabolic Processes

**Figure 5:**
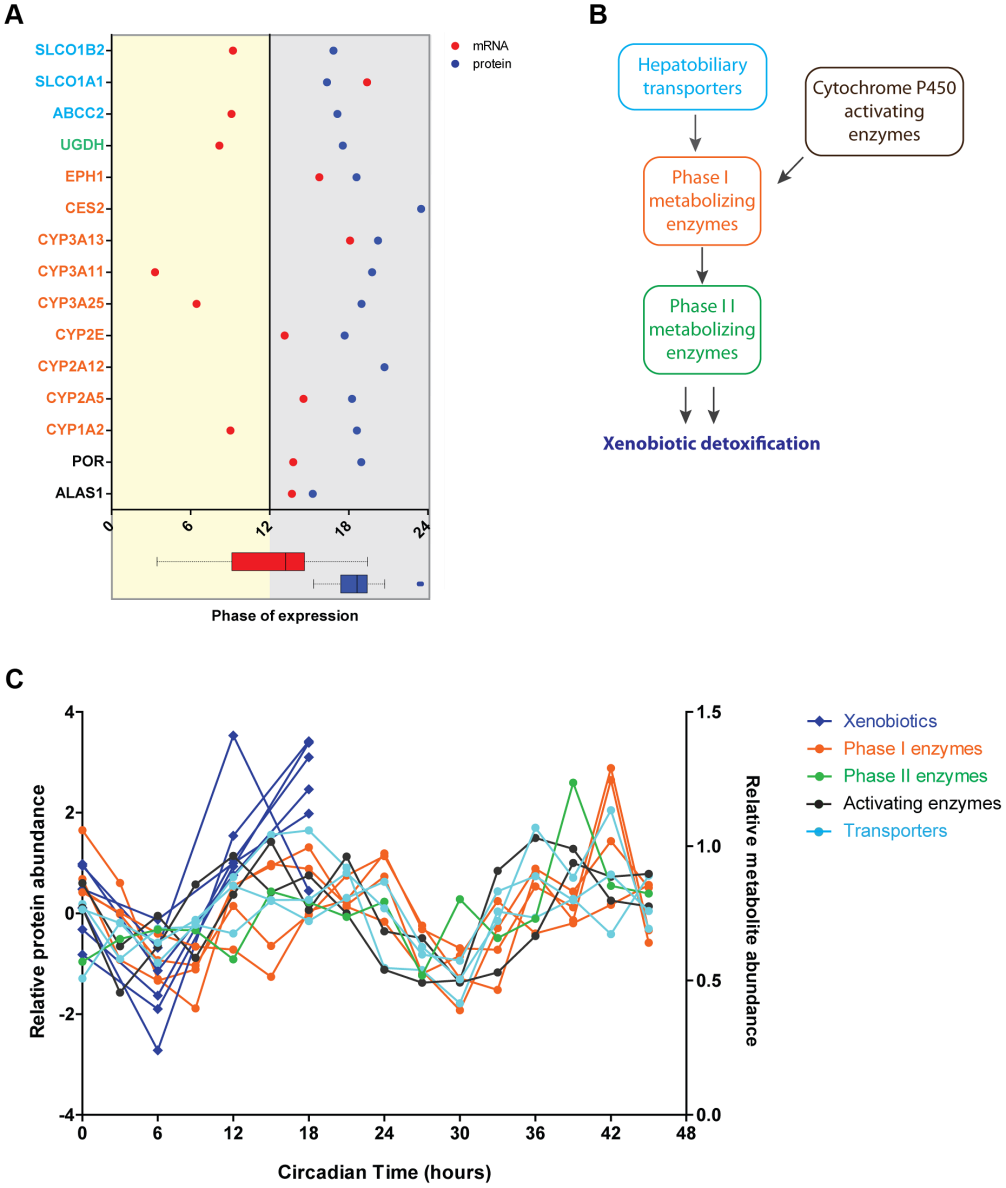
Circadian regulation of metabolism of xenobiotics is large shaped post-transcriptionally. (A) Cycling proteins involved in metabolism of xenobiotics show concomitant phases of abundance in the middle of the night. Graph showed calculated phases of abundance for cycling proteins (blue) involved in the detoxification pathway and their corresponding mRNA (red). Lack of mRNA data in the graph indicates an arrhythmic transcript. At the bottom box plots representing graphically the data of the mRNA (red) and protein (blue) phases plotted in the graph. Protein names are color coded based on their functional role in the pathway as indicated in B. We found 97 proteins involved in this pathway in the total 3131 dataset and 15 proteins in the cycling dataset of 201. (B) Circadian oscillations are found in proteins essential for different stages of the metabolism of xenobiotics. Scheme showing the main two phases of xenobiotic detoxification as well as the contribution of enzymes activating cytochrome P450 proteins essential for phase I and liver transporters. (C) The abundance of rhythmic proteins from the detoxification pathway matches remarkably the levels of xenobiotics in the liver. Expression profiles of cycling proteins (median of Z-scored log 2 normalized ratios for each triplicate) color coded according to their functional role (as in B) and the abundance profile of several xenobiotics n the liver reported by Eckel-Mahan et al. [10].

Having established that the circadian clock drives liver metabolism not only at the level of mRNA but also by precise regulation of the phase of protein abundance, we next examined individual metabolic processes. We found that rhythmic proteins associated to specific functions oscillated with similar phases regardless of whether their transcripts were cycling or, if so, when they were expressed. This implies that circadian post-transcriptional regulation coordinates individual metabolic pathways – something that became especially obvious for the metabolism of xenobiotics. Phases of abundance of crucial components of this pathway tightly clustered at the end of the night while there was no obvious coordination in the phases of the cycling mRNAs (Figure 5A). Moreover, temporal coordination of protein abundance of drug metabolism in the liver was not restricted to enzymes involved in the three phases of the detoxification mechanism. Strikingly, it also extended to membrane transporters responsible for the intake of xenobiotic substances from the blood circulation into the liver and/or their subsequent secretion into bile after being conjugated (Figure 5A and 5B). Supporting a metabolic function of this coordinated expression, we found that the abundance profiles of rhythmic enzymes involved in detoxification strongly correlated with the oscillations of xenobiotics levels reported recently in a liver circadian metabolome study [10] (Figure 5C).

A substantial number of proteins involved in complement and coagulation pathways oscillate in abundance, with their phases significantly clustered during the day. Their phases of abundance did not uniformly reflect the cycling patterns of their corresponding transcripts (Figure S3D).

Temporal regulation of gene expression mediated by the circadian transcription factor heat shock factor 1 (HSF1) has been reported for some heat shock proteins [33], however, our study shows for the first time that the protein folding machinery in the mouse liver is also regulated in a circadian manner at the protein level. We found many chaperones undergoing daily oscillations of abundance in the mouse liver with phases remarkably clustered at night. Heat shock proteins present an interesting contrast to the examples described above, in that protein cycles of most of them closely mirror their transcriptional profiles (Figure S3E) showing short time delays between mRNA and protein peaks. Thus, circadian regulation of protein abundance for this pathway seems to be extensively determined at the level of mRNA in contrast to what we observed for the metabolism of xenobiotics and coagulation cascades.

In summary, our analyses indicate that post-transcriptional mechanisms have a large but diverse influence on the oscillation of the proteome essential for different cellular processes. Specifically, the contributions of this circadian post-transcriptional control appear to differ among diverse metabolic pathways.

## Discussion

For technical reasons, it has been difficult to attain high coverage at the level of the proteome. We thus know much more about genome-wide mRNA levels and have used these as proxies of protein abundance. However, recent advances in mass spectrometry and quantitative proteomics now allow studying proteins in a much more comprehensive and high-throughput manner that previously possible. As a result, it is becoming clear that the relationship between transcript and protein abundance is complex. For instance, a recent study quantifying transcripts and proteins in a mammalian cell line suggests that translation rate is the predominant mechanism that regulates cellular protein levels [34].

Circadian control of metabolism has been widely studied based on oscillations of transcripts with the general conclusion that approximately 10% of the transcriptome oscillate daily [3]–[7]. New proteomics methods now allow direct characterization of abundance changes of essential metabolic proteins across the day rather than inferring that from the corresponding RNA levels. Here we presented the first large scale quantitative proteomic approach, aimed at identifying circadian oscillations of protein abundance in the mouse liver and compare them to transcript rhythms. The proportion of the rhythmic liver proteome, around 6%, is notably similar to that reported for the circadian transcriptome in different mouse tissues. An earlier circadian proteome study using 2D gel electrophoresis reported that up to 20% of the assayed soluble proteins in the mouse liver were cycling [13]. This difference to our results, in which we accurately quantified more than 3,000 proteins, is likely due to technical limitations of 2D gel electrophoresis. This is also reflected in the fact that our study, but not the 2D gel study, identified a substantial part of the membrane proteome as cycling.

Our results indicate that circadian clocks coordinate hepatic metabolism and other cellular processes not only by driving transcription but by orchestrating cycles of protein abundance. In particular, we observed significant differences in the phase distribution between cycling transcripts and corresponding proteins (Figure 4B). This denotes a key contribution of circadian post-transcriptional regulatory mechanisms in tuning metabolism.

**Figure 6:**
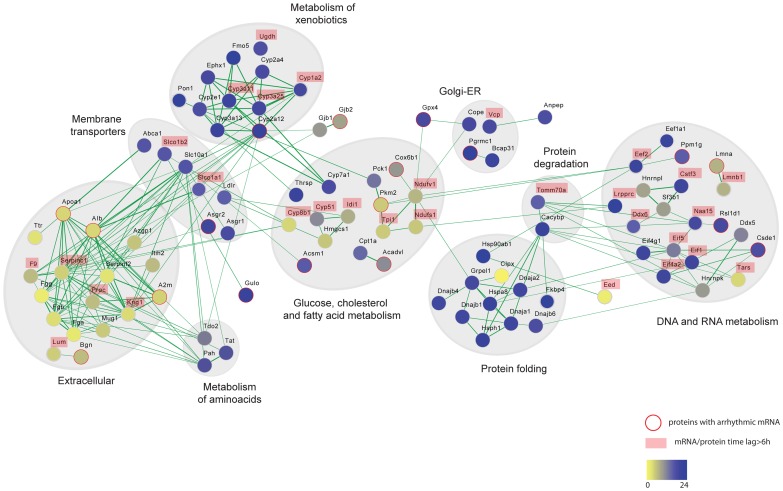
Protein interaction networks of rhythmic liver proteins. Broad range of functional categories can be depicted in the protein interaction network of the circadian liver proteome. The analysis was done using protein interaction information from the STRING database and visualized using Cytoscape. Each node represents a protein that is colored based on the phase of the abundance cycle as indicated in the lower color bar. Red lined nodes designate oscillating proteins which arrhythmic transcripts. Labeled protein names indicate those with time lags between peak of mRNA and protein longer than 6/or protein.

The liver circadian proteome contains proteins involved in a broad range of metabolic processes. We performed a functional or physical interaction network analysis of cycling liver proteins in the STRING database [35], the result of which is visualized with Cytoscape in Figure 6 (see Material and Methods). One of the largest network clusters is comprised of interactions among essential components of xenobiotic metabolism with remarkably coordinated nocturnal phases as we described above. This data indicates that circadian regulation of hepatic xenobiotic detoxification is not only exerted by control of gene expression as previously reported [36]–[38] but moreover by a precise post-transcriptional control that ensures the presence of their essential components at the time of the day when the pathway is metabolically more active. By temporally driving cycles of abundance for detoxifying enzymes with higher levels during the night, the circadian clock can coordinate xenobiotics detoxification in the mouse liver to cycles of metabolic needs, ensuring proper detoxification during the nocturnal phase when mice are feeding and thus ingesting the majority of xenobiotics. This hypothesis is independently supported by recent metabolomics data [10], which shows that the level of toxic metabolites in the liver cycle in accordance with the protein rhythms characterized here. Understanding abundance and activity cycles of detoxifying enzymes is an essential prerequisite for the determination of temporal variations of therapeutic responses and associated toxic effects both ultimately crucial for proper chronotherapy.

Another prominent interaction network consists of extracellular proteins that are synthesized in the liver (Figure 6). Some studies have reported circadian variations in plasma levels of hemostatic factors that seemed to be preceded by liver oscillations of their respective mRNAs [39]–[41]. However it is not clear how circadian regulation of hepatic metabolism contributes to hemostasis. The fact that plasma components synthesized in the liver show hepatic protein cycles indicates that the daily oscillations of essential hemostatic components in the plasma may reflect, at least in part, hepatic protein rhythms. Supporting such observations, hemostatic variables have been shown to undergo circadian changes in humans [42]. For instance, predisposition towards clotting in the morning, due to increased levels of platelet aggregation and blood coagulation, has been associated with the higher incidence of myocardial infarctions. In contrast, fibrinolysis is enhanced in the evening concomitant with higher levels of thrombolytic factors [43]–[45]. An additional aspect of liver metabolism that seems to be shaped by circadian post-transcriptional control is the glucose and fatty acid metabolism. We see circadian rhythms of protein abundance in several essential enzymes of these pathways, many of them lacking temporal regulation at the level of transcription or showing protein phases almost in anti-phase to their cycling transcript (Figure 6).

In addition to specific metabolic processes we found coordinated rhythms of abundance in liver proteins involved in more general cellular functions, such as protein folding, vesicle-mediated transport as well as DNA and RNA metabolism (Figure 6). Thus, an important node in our interaction network comprised a large number of chaperones oscillating with night phases, possibly indicating higher demand for protein folding or quality control at this time of day (Figure S3E and Figure 6). Similarly, many key components of vesicle trafficking oscillate in abundance in the mouse live with synchronized phases at night, unlike their transcripts (Figure 6). This is the case of several RAB GTPases such as RAB1, an essential factor for ER-Golgi transport [46] which transcript is arrhythmic, as well as for RAB10 and RAB14, both involved in the endocytic pathway and the late endosome-associated RAB7 protein (Figure S4A, S4B and S4C). Protein rhythms with peak of abundance during the night were also observed for the small GTPases SAR1 and the ADP-ribosylation factor 5, ARF5 (Figure S4B and S4C). While SAR1 controls the association of COPII with ER membranes [47], the conserved GTPase ARF5 associated with coatomer, constituting the minimal cytosolic machinery leading to COPI vesicle formation from Golgi membranes [48]. This data indicates that key proteins involved in both ER and Golgi vesicle formation are under circadian regulation. Moreover, rhythms of protein abundance can be found in the epsilon subunit of the coatomer, COPE (Figure S4B), a coating complex crucial for intra-Golgi trafficking, retrograde Golgi-to-ER transport of dilysine-tagged proteins as well as for the processing, activity and endocytic recycling of LDL receptor (LDLR) [49]. Therefore rhythms of COPE and its associated GTPase ARF5 could determine cycles of recycling for essential hepatic receptors as LDLR as well as the liver specific C-type lectin asialoglycoprotein receptors ASGPR1 and ASGPR2 (all rhythmic) ensuring proper expression during the night when the liver receives most of the metabolic signals in mice.

The liver is a key player in the regulation of cholesterol levels. It synthesizes cholesterol for export to other cells and removes cholesterol from the circulation by converting it to bile salts and excreting it into the bile. Additionally, the liver produces the various lipoproteins involved in transporting cholesterol and lipids throughout the body. While total cholesterol in plasma does not seem to oscillate daily, high and low density lipoprotein cholesterol (HLD and LDL), show circadian rhythms in plasma with a through at the onset of the dark phase [50], [51]. LDL is the major transporter of cholesterol in plasma and in humans proper LDLR-mediated hepatic cholesterol removal plays a crucial role in atherosclerosis prevention. Ldlr gene expression is reported to be circadian in rat liver [50] and in a human hepatocarcinoma cell line via SREBP as well as CLOCK/BMAL1 direct promoter activation [51]. Our study shows for the first time that the LDLR also exhibits daily cycles of protein abundance in the mouse liver with at least two-fold higher levels in the middle of the night (Figure S4D). Interestingly the peak of the mouse hepatic LDLR correlates with lower LDL plasma levels, similar to what has been reported at the transcript level in rat livers [50]. The ABC transporter ABCA1 is crucial for maintaining plasma HDL levels due to its essential role in assembling cholesterol, phospholipids and APOA1 into HDL. It is also rhythmic in the mouse liver with maximum presence during the night (Figure S4D) correlating with high levels of lipids in plasma [52]. Two major lipoproteins APOA1 and APOOL also oscillated in abundance in the mouse liver (Figure S4E) while lack rhythms at the transcripts level. Their daytime peaks of expression are though in anti-phase to the reported peak of APOB in plasma [52]. In addition to dietary intake the other source of cholesterol is de novo synthesis in hepatocytes which is under negative feedback regulation: increased cholesterol in the cell decreases the expression and activity of HMG-CoA reductase (HMGR), as well as the expression of the lanosterol 14 -demethylase, Cyp51, both essential enzymes in cholesterol biosynthesis and intermediate metabolites [53]. Although we did not obtained quantitative values for HMGR our analysis identified circadian cycles of protein abundance in the mouse liver for several key enzymes involved in cholesterol and bile acid synthesis such as HMG-CoA synthase 1 (HMGCS1), isopentenyl-diphosphate delta-isomerase 1 (IDI1), CYP51, CYP7A1 and CYP8B1 (Figure 6 and S4F). The oscillation of CYP7A1 is concomitant with the rhythm of its mRNA, however, we observed relative long time delays between the peaks of abundance for the other proteins compared to their respective transcript (Table S4). In particular, CYP51 shows maximum levels at the onset of the night completely in anti-phase to its transcript. Together our data indicates that hepatic circadian control of cholesterol homeostasis and bile acids biosynthesis is not only driven at the level of transcription [9] but additionally defined post-transcriptionally. Moreover, circadian oscillations in the levels of these enzymes, most of them localized in the ER membrane, could be linked to the described circadian dilation of the ER in hepatocytes [54] which is an indication of ER stress. The ER responds to the stress by activating the unfolded protein response (UPR) to reduce the accumulation of unfolded proteins. Concordant with this the circadian proteome has an overrepresentation of proteins with nocturnal phases involved in the protein processing in ER (Figure S2B and S2D). Moreover, it has recently been established that there is a connection between the metabolite-induced activation of the UPR, hepatic transformation of metabolites and the circadian clock controlled feeding behavior and all of these are essential for proper liver metabolism [55].

Another large node in our interaction network is comprised of cycling proteins involved in DNA and RNA metabolism (Figure 6). Among them many essential factors of the protein translation machinery oscillated in their abundance. For example the translation initiation factors EIF1, E1F4A2, EF4G1 and EIF5 showed concomitant rhythmic profiles of protein abundance across the two analyzed cycles (Figure S5A). Furthermore, we observed cycles of abundance of two indispensable components of protein translation elongation, EEF2 and EEF1A1, in phase with the translation initiation elements (Figure S5B). Our data thus suggests rhythms of translation by means of protein abundance of indispensable pathway components. In support of this hypothesis, a study that appeared after our analysis was finished showed that the circadian clock influences the temporal translation of a group of mRNAs by regulating the expression and activation of essential translation factors [15]. In particular, we observed that the cycles of abundances of these factors with peaks at the middle of the night, between CT19 and CT20 (Table S2), strongly correlate with the time of the day when 70% of the circadian translationally regulated genes are found in the polysomal fraction [15]. Furthermore, most of the mRNAs temporal translated during the night are involved in ribosome biogenesis correlating with our phase enrichment results for cycling ribosome proteins (Figure S2C and S2D).

Taken together, our work highlights the importance of circadian post-transcriptional mechanisms in shaping the phase of daily protein oscillations in the liver thus determining cycles of metabolism and physiology. Although the overall role of these mechanisms has been remained elusive, recent studies have emphasized the contribution of temporal post-transcriptional regulation on the circadian transcriptome [16], [18], [32]. Only up to approximately 30% of cycling mRNAs also showed rhythms in transcription, implying that post-transcriptional regulation largely defines the oscillating mRNA pool. A potential mechanism for clock-regulated post-transcriptional regulation of mRNA is reported by Schibler and colleagues, showing a temperature dependent cycling of cold–inducible RNA binding protein (CIRP) binding to and regulating the amplitude of transcripts from several core circadian components, including Clock [18]. By assaying protein rhythms, we move a step further along the gene expression program. We conclude that although around 80% of the oscillating proteins are associated with rhythmic transcripts the phases of many of them are uniquely tuned post-transcriptionally, suggesting a temporal mechanistic heterogeneity in this molecular process.

This study demonstrates that the mouse liver proteome is extensively regulated by the circadian clock, with about 6% of proteins in our dataset significantly oscillating with peaks at a variety of phases. The distribution of phases emphasizes the complexity of circadian post-transcriptional mechanisms. Protein oscillations ultimately govern circadian rhythms of cellular and metabolic processes essential for the fitness of the organism. Our findings point to clock regulation of many more individual proteins and entire pathways, elucidating new networks that may be conferring previously uncharacterized rhythms in metabolism and physiology. In addition to protein cycles of abundance, post-translational modifications are known to have pivotal roles in the clock molecular machinery. The same proteomics technologies employed here can also be used to quantify post-translational modifications and thereby investigate to what extent and how they also drive global circadian patterns.

## Materials and Methods

### Animals and Tissue Collection

All mice were bred and maintained in the animal facility of the Max Planck Institute of Biochemistry according to institutional guidelines and all animal experiments were approved by the government agencies of Oberbayern. Eight-week-old C57BL/6 mice were house in light-tight boxes with free access to food and water and entrained to a 12–12 h light-dark schedule for ten days before being transfer to complete darkness. After one day in constant darkness, mice were sacrificed at 3 h intervals over two days. Prior to liver excision, mice were perfused with ice-cold PBS to remove blood content. Livers were then quickly frozen in liquid nitrogen followed by storage at −80°C. SILAC mice [23] were kept in the same conditions; two animals were sacrificed in anti-phase at CT3 and CT15, respectively, and livers excised as described above.

### Protein Sample Preparation

Protein extracts from mouse livers were obtained as previously described [56], [57]. Briefly, homogenization of 1 mg of liver was done in 1 ml of 0.1 mM Tris-HCl pH 7.6 supplemented with complete protease and phosphatase inhibitor cocktails (Roche) using an Ultra Turbax blender (IKA) at maximum speed at 4°C for 30–60 seconds. Sodium dodecyl sulfate (SDS) and dithiothreitol (DTT) were added to the homogenates to a final concentration of 4% and 0.1 mM, respectively, followed by brief sonication to reduce viscosity. After 5 min incubation at 95°C the mixture was then cleared by centrifugation at 16,000× g at room temperature for 10 min. Protein content was determined by measurements of tryptophan fluorescence as previously described [56]. Sixteen protein extract pools, corresponding to the samples collected at different circadian times, were obtained by mixing equal amounts of protein liver extracts from each of the four mice sacrificed at any given time point. Similarly the SILAC protein liver mix was obtained by adding equal amounts of the protein extracts obtained from the two SILAC liver samples collected in anti-phase.

### Protein Digestion and Anion Exchange Peptide Separation

For sample preparation we used 100 µg of protein extract from each circadian time pool mixed with 100 µg of SILAC protein pool. The protein mixes were concentrated in 30 k Microcon filtration devices (Millipore) to a final volume of 30 µl and then processed by the FASP procedure [57]. Briefly, the samples were mixed with 0.2 ml of 8 M urea in 0.1 M Tris/HCl pH 8.5 (UA), loaded into 30 k Microcon filtration devices (Millipore) and centrifuged at 14,000× g for 15 min. The concentrates were diluted in the devices with 0.2 ml of UA solution and centrifuged again. After centrifugation the concentrates were mixed with 0.1 ml of 50 mM iodoacetamide in UA solution and incubated in the dark at room temperature for 30 min. After centrifugation for 15 min the concentrate was diluted with 0.2 ml UA solution and concentrated again by centrifugation. This step was repeated twice. Next, the concentrate was diluted with 0.1 ml of 40 mM NaHCO_3_ and concentrated again twice. Subsequently, 2 µg of Lysyl Endopeptidase (Wako Chemicals) in 40 µl of 40 mM NaHCO3 was added to the filter and the samples were incubated at room temperature overnight. The peptides were collected by centrifugation of the filter followed by two additional 30 µl washes with 40 mM NaHCO_3_. The concentration of peptides was determined by UV-spectrometry using an extinction coefficient of 1.1 for 0.1% (g/l) solution at 280 nm.

Peptides were dissolved in 200 µL in Britton & Robinson buffer composed of 20 mM CH_3_COOH, 20 mM H_3_PO_4_, and 20 mM H_3_BO_3_, and NaOH, pH 11. The peptides were separated by a pipette-based anion exchanger method [57]. Briefly, the pipette based column was assemble by stacking 6 layers of a 3M Empore Anion Exchange disk (Varian, 1214-5012) into a 200 µl micropipette tip. For column equilibration and elution of fractions Britton & Robinson buffer titrated with NaOH to the desired pH was used. Peptides were loaded at pH 11 and fractions were subsequently eluted with buffer solutions of pH 8, 6, 5, 4, and 3, respectively.

### Mass Spectrometric Analysis and Data Processing

All mass spectrometric (MS) experiments were performed on a nanoflow HPLC system (Proxeon Biosystems, now Thermo Fisher Scientific) connected to a hybrid LTQ-Orbitrap (Thermo Fisher Scientific, Bremen, Germany), equipped with a nanoelectrospray ion source (Proxeon Biosystems, now Thermo Fisher Scientific). Peptide mixtures were separated by reversed phase chromatography using in-house-made C_18_ microcolumns with a diameter of 75 µm packed with ReproSil-Pur C18-AQ 3-µm resin (Dr. Maisch GmbH, Ammerbuch-Entringen, Germany) in 4 hours LC gradient from 3% to 75% acetonitrile in 0.5% acetic acid at a flow rate of 200 nl/min and directly electrosprayed into the mass spectrometer. The LTQ-Orbitrap was operated in the positive mode to simultaneously measure full scan MS spectra (from m/z 300–1650) in the Orbitrap analyzer at resolution R = 60 000 following isolation and fragmentation of the ten most intense ions in the LTQ part by collision-induced dissociation. Raw MS files were processed with MaxQuant (version. 1.1.1.9), a freely available software suite. Peak list files were searched by the ANDROMEDA a search engine, incorporated into the MaxQuant framework [58], against the IPI-mouse (version 3.68) containing both forward and reversed protein sequences. Initial maximum precursor and fragment mass deviations were set to 7 ppm and 0.5 Da, respectively, but MaxQuant achieved sub-ppm mass accuracy for the majority of peptide precursors. The search included variable modifications for oxidation of methionine, protein N-terminal acetylation and carbamidomethylation as fixed modification. Peptides with at least six amino acids were considered for identification specifying as enzyme LysC allowing N-terminal cleavage to proline. The false discovery rate, determined by searching a reverse database, was set at 0.01 for both peptides and proteins. Identification across different replicates and adjacent fractions was achieved by enabling matching between runs option in MaxQuant within a time window of 2 minutes. Quantification of SILAC pairs was performed by MaxQuant with standard settings using a minimum ratio count of 2.

### Bioinformatics Analysis

All bioinformatic analyses were performed with the Perseus software (http://www.perseus-framework.org/). To determine the subset of cycling proteins, each protein expression profile is fitted to a cosine with a fixed period of 23.6 h and the amplitude and phase as free parameters. Profiles are ranked by their variance ratio. This is the part of the variance explained by the fit divided by the contribution to the variance that is not accounted for by the fit. Based on this ranking we determine a permutation-based false discovery rate by repeating the same procedure 1,000 times on the same profiles but with scrambled time labels, except for the technical replicates that were preserved. This permutation based procedure is similar to the one applied to FDR calculations for differential expression analysis in [59]. Hierarchical clustering was done in a phase-preserving way by restricting the order of elements to the one determined by the output of the cosine model-based fitting. During the growth of the tree in hierarchical clustering, only those links were permitted that conserve this order. While obviously the order of the terminal branches is not an outcome of the algorithm, the cluster structure is still a non-trivial result of the clustering.

### Principal Component Analysis (PCA)

A standard PCA analysis was performed in the Perseus software. The expression data matrix has protein groups as rows and samples as columns and contains logarithms of ratios. Missing values were imputed by drawing random numbers from a normal distribution to simulate signals from low abundant proteins. The width parameter of this normal distribution was chosen as 0.3 of the standard deviation of all measured values and the center was shifted towards low abundance by 1.8 times this standard deviation. These parameters were empirically determined to result in good performance over many different proteomics data sets. The row means were subtracted from the matrix. Then the PCA was performed by singular value decomposition.

### Western Blotting

Total cell protein extracts were prepared as mentioned above and 50 µg of protein was used for western blotting performed according to standard protocols. Antibodies used were PER21A from Alpha Diagnostics International, RAB1 from Sigma-Aldrich, SARA1; GAPDH and RAB10 from Cell Signaling. The density of the bands obtained in the western blots was calculated with the freely available gel analyzer program ImageJ.

### RNA Extraction and Quantitative Real-Time PCR

RNA extraction from liver was done using the RNeasy kit according to manufacturer's protocol (Qiagen). cDNA was synthesized from 2 µg of liver total RNA using First Strand cDNA Synthesis kit following the supplier's instruction (Fermentas). Quantitative PCR reactions were done by amplifying ten per cent of the cDNA with Sybr Green master mix (Applied Bioscience) on a CFX96 Real Time System (BioRad). Mean values were calculated from triplicate PCR assays for each sample and normalized to those obtained for *Gadph* transcript.

### Interaction Network Analysis

Interaction network analysis of the cycling proteome was performed with the STRING search tool (version 9.0) using medium to high confidence (0.5–0.7) and with co-expression and experiments as active prediction methods. Using these parameters we obtained interaction scores for approximately 70% of the liver rhythmic proteins. Data visualization was done with Cytoscape 2.8.2 where we then combined interaction scores with phases of protein abundance.

Data availability: The mass spectrometry proteomics data have been deposited to the ProteomeXchange Consortium ( http://proteomecentral.proteomexchange.org ) via the PRIDE partner repository [60] with the dataset identifier PXD000601.

## Supporting Information

Figure S1 Proper entrainment of the mice. (A) Quantitative reverse-transcriptase PCR assay showing the temporal expression profiles of *Bmal1* (left) *and Per2* (right) mRNA in the liver samples used for the proteomic analysis. Shown are mean and S.E.M.; N = 3. Data were normalized to *Gapdh* mRNA expression. (B) Western blots detecting PER2 protein in the liver samples used for the proteomic analysis. Loading control is shown with anti-GAPDH antibody. Specific band is indicated with an asterisk. (C) Scatter plot showing the correlation between q-values calculated with our Perseus package and JTK_cycle for the protein dataset containing valid values in all measured samples (1888). Blue dots correspond to common statistical significant cycling proteins after using a cut off of q-value<0.33 (dashed bars) in both methods. Green dots are those statistical significant in only one of the two methods. The horizontal and vertical effect observed in the lower values is due to the discretization of the permutation-based calculation of the q-value. (D) Plot showing the correlation of phases estimated with Perseus and JTK_ cycle of the cycling proteome. (E) Histograms show the distribution of fold change of acetylated (left panel), conjugated to ubiquitin-like modifier proteins (middle panel) and glycosilated (right panel) proteins (green) in the total cycling (blue) liver proteome.
(TIF)

Figure S2 Phase dependent enrichment analysis of the liver circadian proteome. (A–B) Distribution of phases of abundance for all cycling proteins (blue) and for those annotated in the indicated KEGG category (red) which shown statistical significant phase enrichment (Benjamini Hochberg FDR<0.05) at day (A) or night (B). (C–D) Protein annotations from Gene Ontology Cellular Component (GOCC) (C) and KEGG pathways (D) enriched in a phase dependent manner in the cycling proteome plotted based on their calculated phase and p-value from the enrichment analysis.
(TIF)

Figure S3 Characteristic time delay between the phase of rhythmic proteins and their corresponding transcripts. (A) Scatter plot showing the correlation between the calculated q-values for cycling proteins and mRNAs in the total quantified dataset (3132). The horizontal and vertical effect observed in the lower values is due to the discretization of the permutation-based calculation of the q-value. Red dots correspond to common statistical significant cycling proteins after using a cut off of q-value<0.33 (dashed bars) in both datasets. (B) Scatter plot representing the correlation of phases for cycling proteins and their corresponding mRNAs. (C) Distribution of time delays between peak of mRNA and protein abundances for the liver circadian proteome with rhythmic transcripts. Please note that time dimension is circular with maximum at 24 h as the length of the day. (D–E) Graph shows the phases of liver rhythmic proteins (blue) and their corresponding mRNAs (red) of secreted proteins (D) (11 in 201 cycling versus 29 in 3131 total dataset) as well as chaperones (C) (8 in 201 cycling versus 75 in 3131 total dataset). Lack of mRNA data indicates an arrhythmic transcript. Box plots at the bottom of each graph represent graphically the data of the mRNA (red) and protein (blue) phases.
(TIF)

Figure S4 Coordinated abundance cycles of proteins involved in essential cellular processes. (A) Profiles of protein abundance, across the two sampled cycles, of cycling RAB GTPases in the mouse liver. Represented values correspond to the median of the normalized log2 z-scored ratios for each triplicate and their respective SEM. (B) Phases of cycling proteins (blue) and its correspondent mRNA (red) components of vesicle trafficking. Lack of mRNA data indicates arrhythmic transcript. (C) Western blots detecting RAB1, SARA1 and RAB10 in the samples of the first collected day. Loading control is shown with anti-GAPDH antibody. Numbers at the bottom indicate the relative density of the signal for each specific antibody at each time point normalized by GAPDH signal. (D–F) Profiles of abundance in the mouse liver for cycling proteins essential for cholesterol metabolism: hepatic receptors (D), apolipoproteins (E) and metabolic enzymes (F). Represented values correspond to the median of the normalized log2 z-scored ratios for each triplicate and their respective SEM.
(TIF)

Figure S5 Circadian oscillations of abundances for crucial translation factors in the mouse liver. (A–B) Profiles of protein abundance across the two sampled cycles for essential initiation (A) and elongation (B) translation factors.
(TIF)

Table S1 Total quantified protein dataset with normalized log2 ratios for all measured samples and q-values from the cycling analysis.
(XLSX)

Table S2 Proteins with statistically significant circadian oscillations of abundance in the mouse liver. For each time point values are shown as the median of the triplicates z-scored normalized log2 ratios.
(XLSX)

Table S3 Enriched protein categories among the circadian liver proteome. Underlined categories are represented in Figure 2C and 2D.
(XLSX)

Table S4 Circadian rhythmic proteins with oscillating mRNA in the mouse liver.
(XLSX)

## References

[1] Eckel-Mahan K, Sassone-Corsi P (2009) Metabolism control by the circadian clock and vice versa. Nat Struct Mol Biol 16: 462–467. 19421159 10.1038/nsmb.1595 PMC4073609

[2] Takahashi JS, Hong HK, Ko CH, McDearmon EL (2008) The genetics of mammalian circadian order and disorder: implications for physiology and disease. Nat Rev Genet 9: 764–775. 18802415 10.1038/nrg2430 PMC3758473

[3] Hughes ME, DiTacchio L, Hayes KR, Vollmers C, Pulivarthy S, et al (2009) Harmonics of circadian gene transcription in mammals. PLoS Genet 5: e1000442. 19343201 10.1371/journal.pgen.1000442 PMC2654964

[4] Miller BH, McDearmon EL, Panda S, Hayes KR, Zhang J, et al (2007) Circadian and CLOCK-controlled regulation of the mouse transcriptome and cell proliferation. Proc Natl Acad Sci U S A 104: 3342–3347. 17360649 10.1073/pnas.0611724104 PMC1802006

[5] Panda S, Antoch MP, Miller BH, Su AI, Schook AB, et al (2002) Coordinated transcription of key pathways in the mouse by the circadian clock. Cell 109: 307–320. 12015981 10.1016/s0092-8674(02)00722-5

[6] Storch KF, Lipan O, Leykin I, Viswanathan N, Davis FC, et al (2002) Extensive and divergent circadian gene expression in liver and heart. Nature 417: 78–83. 11967526 10.1038/nature744

[7] Ueda HR, Chen W, Adachi A, Wakamatsu H, Hayashi S, et al (2002) A transcription factor response element for gene expression during circadian night. Nature 418: 534–539. 12152080 10.1038/nature00906

[8] Lamia KA, Storch KF, Weitz CJ (2008) Physiological significance of a peripheral tissue circadian clock. Proc Natl Acad Sci U S A 105: 15172–15177. 18779586 10.1073/pnas.0806717105 PMC2532700

[9] Le Martelot G, Claudel T, Gatfield D, Schaad O, Kornmann B, et al (2009) REV-ERBalpha participates in circadian SREBP signaling and bile acid homeostasis. PLoS Biol 7: e1000181. 19721697 10.1371/journal.pbio.1000181 PMC2726950

[10] Eckel-Mahan KL, Patel VR, Mohney RP, Vignola KS, Baldi P, et al (2012) Coordination of the transcriptome and metabolome by the circadian clock. Proc Natl Acad Sci U S A 109: 5541–5546. 22431615 10.1073/pnas.1118726109 PMC3325727

[11] Fustin JM, Doi M, Yamada H, Komatsu R, Shimba S, et al (2012) Rhythmic nucleotide synthesis in the liver: temporal segregation of metabolites. Cell Rep 1: 341–349. 22832226 10.1016/j.celrep.2012.03.001

[12] Dallmann R, Viola AU, Tarokh L, Cajochen C, Brown SA (2012) The human circadian metabolome. Proc Natl Acad Sci U S A 109: 2625–2629. 22308371 10.1073/pnas.1114410109 PMC3289302

[13] Reddy AB, Karp NA, Maywood ES, Sage EA, Deery M, et al (2006) Circadian orchestration of the hepatic proteome. Curr Biol 16: 1107–1115. 16753565 10.1016/j.cub.2006.04.026

[14] Gachon F, Bonnefont X (2010) Circadian clock-coordinated hepatic lipid metabolism: only transcriptional regulation? Aging (Albany NY) 2: 101–106. 20354271 10.18632/aging.100123 PMC2850146

[15] Jouffe C, Cretenet G, Symul L, Martin E, Atger F, et al (2013) The circadian clock coordinates ribosome biogenesis. PLoS Biol 11: e1001455. 23300384 10.1371/journal.pbio.1001455 PMC3536797

[16] Koike N, Yoo SH, Huang HC, Kumar V, Lee C, et al (2012) Transcriptional Architecture and Chromatin Landscape of the Core Circadian Clock in Mammals. Science 338: 349–354. 22936566 10.1126/science.1226339 PMC3694775

[17] Kojima S, Shingle DL, Green CB (2011) Post-transcriptional control of circadian rhythms. J Cell Sci 124: 311–320. 21242310 10.1242/jcs.065771 PMC3021995

[18] Morf J, Rey G, Schneider K, Stratmann M, Fujita J, et al (2012) Cold-Inducible RNA-Binding Protein Modulates Circadian Gene Expression Posttranscriptionally. Science 338: 379–383. 22923437 10.1126/science.1217726

[19] Aebersold R, Mann M (2003) Mass spectrometry-based proteomics. Nature 422: 198–207. 12634793 10.1038/nature01511

[20] Mallick P, Kuster B (2010) Proteomics: a pragmatic perspective. Nat Biotechnol 28: 695–709. 20622844 10.1038/nbt.1658

[21] Geiger T, Cox J, Ostasiewicz P, Wisniewski JR, Mann M (2010) Super-SILAC mix for quantitative proteomics of human tumor tissue. Nat Methods 7: 383–385. 20364148 10.1038/nmeth.1446

[22] Gouw JW, Krijgsveld J, Heck AJ (2010) Quantitative proteomics by metabolic labeling of model organisms. Mol Cell Proteomics 9: 11–24. 19955089 10.1074/mcp.R900001-MCP200 PMC2808257

[23] Kruger M, Moser M, Ussar S, Thievessen I, Luber CA, et al (2008) SILAC mouse for quantitative proteomics uncovers kindlin-3 as an essential factor for red blood cell function. Cell 134: 353–364. 18662549 10.1016/j.cell.2008.05.033

[24] Zanivan S, Krueger M, Mann M (2012) In vivo quantitative proteomics: the SILAC mouse. Methods Mol Biol 757: 435–450. 21909926 10.1007/978-1-61779-166-6_25

[25] Cox J, Mann M (2008) MaxQuant enables high peptide identification rates, individualized p.p.b.-range mass accuracies and proteome-wide protein quantification. Nat Biotechnol 26: 1367–1372. 19029910 10.1038/nbt.1511

[26] Possidente B, Stephan FK (1988) Circadian period in mice: analysis of genetic and maternal contributions to inbred strain differences. Behav Genet 18: 109–117. 3365193 10.1007/BF01067080

[27] Hughes ME, Hogenesch JB, Kornacker K (2010) JTK_CYCLE: an efficient nonparametric algorithm for detecting rhythmic components in genome-scale data sets. J Biol Rhythms 25: 372–380. 20876817 10.1177/0748730410379711 PMC3119870

[28] Cox J, Mann M (2012) 1D and 2D annotation enrichment: a statistical method integrating quantitative proteomics with complementary high-throughput data. BMC Bioinformatics 13 Suppl 16: S12. 23176165 10.1186/1471-2105-13-S16-S12 PMC3489530

[29] Rey G, Cesbron F, Rougemont J, Reinke H, Brunner M, et al (2011) Genome-wide and phase-specific DNA-binding rhythms of BMAL1 control circadian output functions in mouse liver. PLoS Biol 9: e1000595. 21364973 10.1371/journal.pbio.1000595 PMC3043000

[30] Kojima S, Sher-Chen EL, Green CB (2012) Circadian control of mRNA polyadenylation dynamics regulates rhythmic protein expression. Genes Dev 26: 2724–2736. 23249735 10.1101/gad.208306.112 PMC3533077

[31] Le Martelot G, Canella D, Symul L, Migliavacca E, Gilardi F, et al (2012) Genome-wide RNA polymerase II profiles and RNA accumulation reveal kinetics of transcription and associated epigenetic changes during diurnal cycles. PLoS Biol 10: e1001442. 23209382 10.1371/journal.pbio.1001442 PMC3507959

[32] Menet JS, Rodriguez J, Abruzzi KC, Rosbash M (2012) Nascent-Seq reveals novel features of mouse circadian transcriptional regulation. Elife 1: e00011. 23150795 10.7554/eLife.00011 PMC3492862

[33] Reinke H, Saini C, Fleury-Olela F, Dibner C, Benjamin IJ, et al (2008) Differential display of DNA-binding proteins reveals heat-shock factor 1 as a circadian transcription factor. Genes Dev 22: 331–345. 18245447 10.1101/gad.453808 PMC2216693

[34] Schwanhausser B, Busse D, Li N, Dittmar G, Schuchhardt J, et al (2011) Global quantification of mammalian gene expression control. Nature 473: 337–342. 21593866 10.1038/nature10098

[35] Szklarczyk D, Franceschini A, Kuhn M, Simonovic M, Roth A, et al (2011) The STRING database in 2011: functional interaction networks of proteins, globally integrated and scored. Nucleic Acids Res 39: D561–568. 21045058 10.1093/nar/gkq973 PMC3013807

[36] Claudel T, Cretenet G, Saumet A, Gachon F (2007) Crosstalk between xenobiotics metabolism and circadian clock. FEBS Lett 581: 3626–3633. 17451689 10.1016/j.febslet.2007.04.009

[37] Gachon F, Olela FF, Schaad O, Descombes P, Schibler U (2006) The circadian PAR-domain basic leucine zipper transcription factors DBP, TEF, and HLF modulate basal and inducible xenobiotic detoxification. Cell Metab 4: 25–36. 16814730 10.1016/j.cmet.2006.04.015

[38] Howell SR, Klaassen C (1991) Circadian variation of hepatic UDP-glucuronic acid and the glucuronidation of xenobiotics in mice. Toxicol Lett 57: 73–79. 2048163 10.1016/0378-4274(91)90121-l

[39] Bertolucci C, Cavallari N, Colognesi I, Aguzzi J, Chen Z, et al (2008) Evidence for an overlapping role of CLOCK and NPAS2 transcription factors in liver circadian oscillators. Mol Cell Biol 28: 3070–3075. 18316400 10.1128/MCB.01931-07 PMC2293078

[40] Bertolucci C, Pinotti M, Colognesi I, Foa A, Bernardi F, et al (2005) Circadian rhythms in mouse blood coagulation. J Biol Rhythms 20: 219–224. 15851528 10.1177/0748730405275654

[41] Ohkura N, Oishi K, Sakata T, Kadota K, Kasamatsu M, et al (2007) Circadian variations in coagulation and fibrinolytic factors among four different strains of mice. Chronobiol Int 24: 651–669. 17701678 10.1080/07420520701534673

[42] Muller OM, Gerber HB (1985) Circadian changes of the rat pancreas acinar cell. A quantitative morphological investigation. Scand J Gastroenterol Suppl 112: 12–19. 10.3109/00365528509092208 3859913

[43] Decousus H, Boissier C, Perpoint B, Page Y, Mismetti P, et al (1991) Circadian dynamics of coagulation and chronopathology of cardiovascular and cerebrovascular events. Future therapeutic implications for the treatment of these disorders? Ann N Y Acad Sci 618: 159–165. 2006785 10.1111/j.1749-6632.1991.tb27244.x

[44] Kurnik PB (1996) Practical implications of circadian variations in thrombolytic and antithrombotic activities. Cardiol Clin 14: 251–262. 8724557 10.1016/s0733-8651(05)70278-2

[45] Montagnana M, Salvagno GL, Lippi G (2009) Circadian variation within hemostasis: an underrecognized link between biology and disease? Semin Thromb Hemost 35: 23–33. 19308890 10.1055/s-0029-1214145

[46] Stenmark H (2009) Rab GTPases as coordinators of vesicle traffic. Nat Rev Mol Cell Biol 10: 513–525. 19603039 10.1038/nrm2728

[47] Matsuoka K, Orci L, Amherdt M, Bednarek SY, Hamamoto S, et al (1998) COPII-coated vesicle formation reconstituted with purified coat proteins and chemically defined liposomes. Cell 93: 263–275. 9568718 10.1016/s0092-8674(00)81577-9

[48] Popoff V, Langer JD, Reckmann I, Hellwig A, Kahn RA, et al (2011) Several ADP-ribosylation factor (Arf) isoforms support COPI vesicle formation. J Biol Chem 286: 35634–35642. 21844198 10.1074/jbc.M111.261800 PMC3195608

[49] Beck R, Rawet M, Wieland FT, Cassel D (2009) The COPI system: molecular mechanisms and function. FEBS Lett 583: 2701–2709. 19631211 10.1016/j.febslet.2009.07.032

[50] Balasubramaniam S, Szanto A, Roach PD (1994) Circadian rhythm in hepatic low-density-lipoprotein (LDL)-receptor expression and plasma LDL levels. Biochem J 298 (Pt 1) 39–43. 8129729 10.1042/bj2980039 PMC1137980

[51] Lee YJ, Han DH, Pak YK, Cho SH (2012) Circadian regulation of low density lipoprotein receptor promoter activity by CLOCK/BMAL1, Hes1 and Hes6. Exp Mol Med 44: 642–652. 22913986 10.3858/emm.2012.44.11.073 PMC3509181

[52] Pan X, Hussain MM (2007) Diurnal regulation of microsomal triglyceride transfer protein and plasma lipid levels. J Biol Chem 282: 24707–24719. 17575276 10.1074/jbc.M701305200

[53] Rezen T, Rozman D, Pascussi JM, Monostory K (2011) Interplay between cholesterol and drug metabolism. Biochim Biophys Acta 1814: 146–160. 20570756 10.1016/j.bbapap.2010.05.014

[54] Chedid A, Nair V (1972) Diurnal rhythm in endoplasmic reticulum of rat liver: electron microscopic study. Science 175: 176–179. 5008436 10.1126/science.175.4018.176

[55] Cretenet G, Le Clech M, Gachon F (2010) Circadian clock-coordinated 12 Hr period rhythmic activation of the IRE1alpha pathway controls lipid metabolism in mouse liver. Cell Metab 11: 47–57. 20074527 10.1016/j.cmet.2009.11.002

[56] Wisniewski JR, Nagaraj N, Zougman A, Gnad F, Mann M (2010) Brain phosphoproteome obtained by a FASP-based method reveals plasma membrane protein topology. J Proteome Res 9: 3280–3289. 20415495 10.1021/pr1002214

[57] Wisniewski JR, Zougman A, Mann M (2009) Combination of FASP and StageTip-based fractionation allows in-depth analysis of the hippocampal membrane proteome. J Proteome Res 8: 5674–5678. 19848406 10.1021/pr900748n

[58] Cox J, Neuhauser N, Michalski A, Scheltema RA, Olsen JV, et al (2011) Andromeda: a peptide search engine integrated into the MaxQuant environment. J Proteome Res 10: 1794–1805. 21254760 10.1021/pr101065j

[59] Tusher VG, Tibshirani R, Chu G (2001) Significance analysis of microarrays applied to the ionizing radiation response. Proc Natl Acad Sci U S A 98: 5116–5121. 11309499 10.1073/pnas.091062498 PMC33173

[60] Vizcaino JA, et al The Proteomics Identifications (PRIDE) database and associated tools: status in 2013. Nucleic Acids Res 2013 Jan 1;41 (D1) D1063–9. 23203882 10.1093/nar/gks1262 PMC3531176

