# Supplementary figures and images for: In-Vivo Quantitative Proteomics Reveals a Key Contribution of Post-Transcriptional Mechanisms to the Circadian Regulation of Liver Metabolism
Source: PLoS Genet. 2014 Jan 2;10(1):e1004047. doi: 10.1371/journal.pgen.1004047 (PMC3879213; doi:10.1371/journal.pgen.1004047)

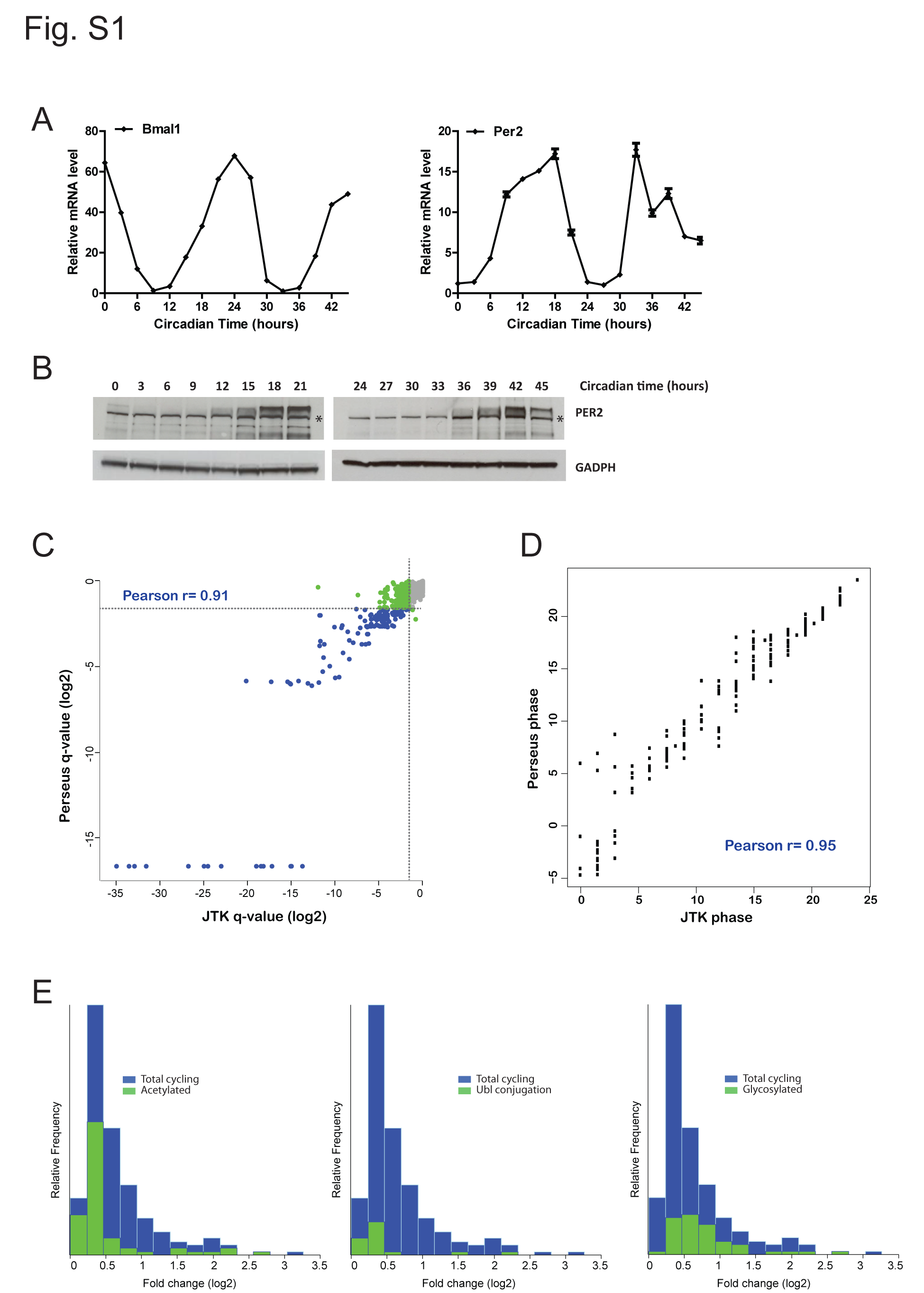

Supplement: Figure S1 — Proper entrainment of the mice. (A) Quantitative reverse-transcriptase PCR assay showing the temporal expression profiles of Bmal1 (left) and Per2 (right) mRNA in the liver samples used for the proteomic analysis. Shown are mean and S.E.M.; N = 3. Data were normalized to Gapdh mRNA expression. (B) Western blots detecting PER2 protein in the liver samples used for the proteomic analysis. Loading control is shown with anti-GAPDH antibody. Specific band is indicated with an asterisk. (C) Scatter plot showing the correlation between q-values calculated with our Perseus package and JTK_cycle for the protein dataset containing valid values in all measured samples (1888). Blue dots correspond to common statistical significant cycling proteins after using a cut off of q-value<0.33 (dashed bars) in both methods. Green dots are those statistical significant in only one of the two methods. The horizontal and vertical effect observed in the lower values is due to the discretization of the permutation-based calculation of the q-value. (D) Plot showing the correlation of phases estimated with Perseus and JTK_ cycle of the cycling proteome. (E) Histograms show the distribution of fold change of acetylated (left panel), conjugated to ubiquitin-like modifier proteins (middle panel) and glycosilated (right panel) proteins (green) in the total cycling (blue) liver proteome. (TIF) [file pgen.1004047.s001.tif]

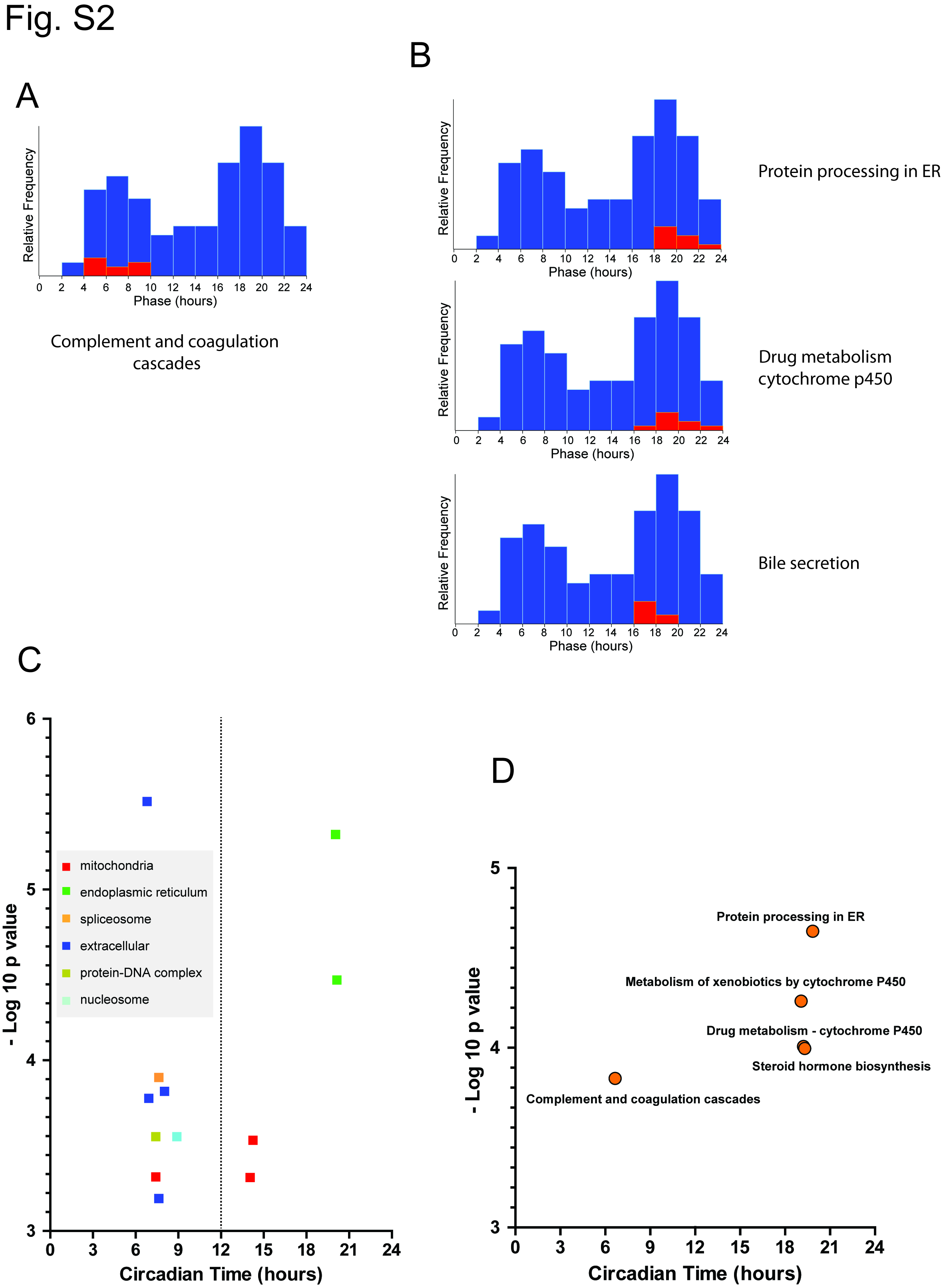

Supplement: Figure S2 — Phase dependent enrichment analysis of the liver circadian proteome. (A–B) Distribution of phases of abundance for all cycling proteins (blue) and for those annotated in the indicated KEGG category (red) which shown statistical significant phase enrichment (Benjamini Hochberg FDR<0.05) at day (A) or night (B). (C–D) Protein annotations from Gene Ontology Cellular Component (GOCC) (C) and KEGG pathways (D) enriched in a phase dependent manner in the cycling proteome plotted based on their calculated phase and p-value from the enrichment analysis. (TIF) [file pgen.1004047.s002.tif]

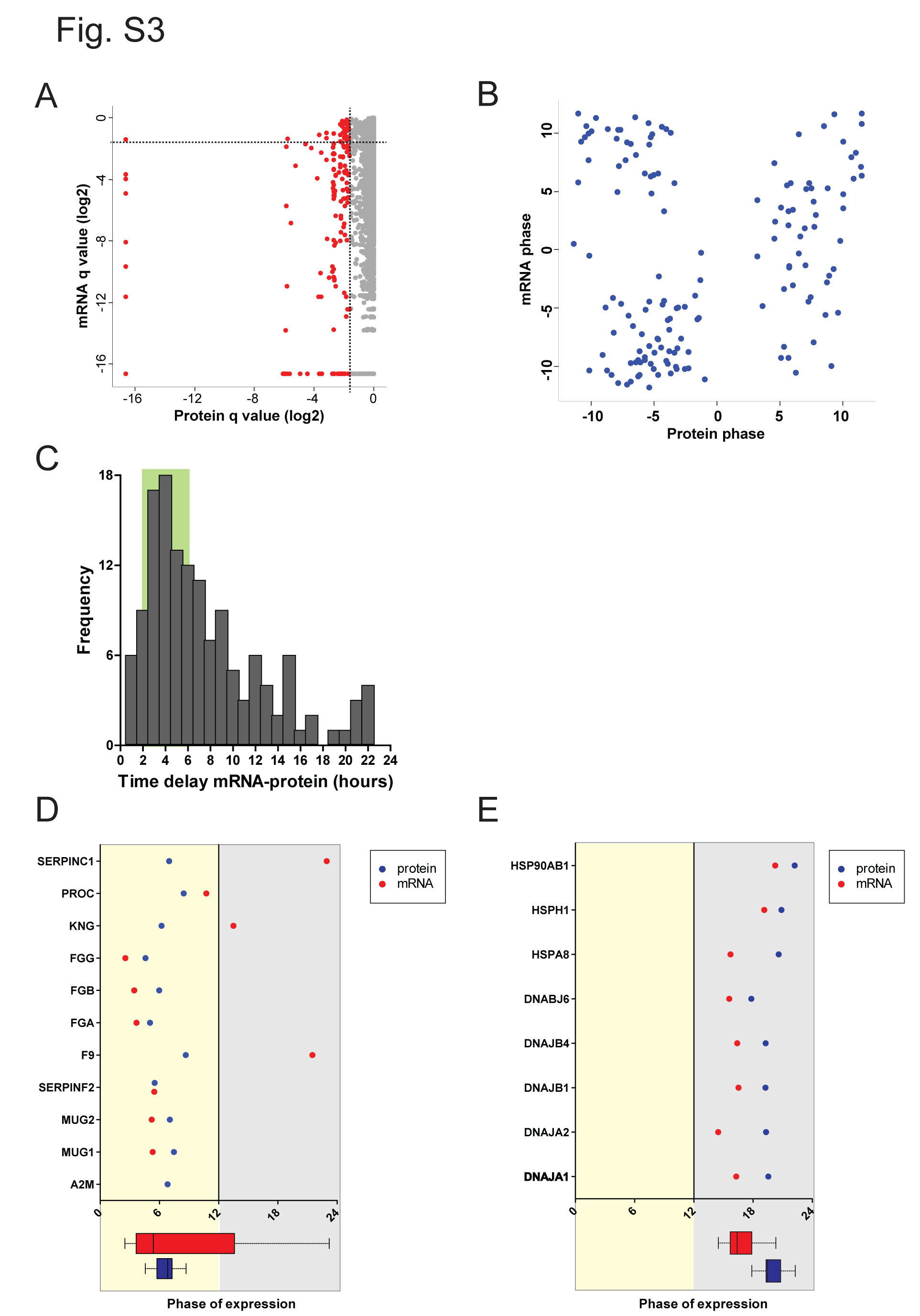

Supplement: Figure S3 — Characteristic time delay between the phase of rhythmic proteins and their corresponding transcripts. (A) Scatter plot showing the correlation between the calculated q-values for cycling proteins and mRNAs in the total quantified dataset (3132). The horizontal and vertical effect observed in the lower values is due to the discretization of the permutation-based calculation of the q-value. Red dots correspond to common statistical significant cycling proteins after using a cut off of q-value<0.33 (dashed bars) in both datasets. (B) Scatter plot representing the correlation of phases for cycling proteins and their corresponding mRNAs. (C) Distribution of time delays between peak of mRNA and protein abundances for the liver circadian proteome with rhythmic transcripts. Please note that time dimension is circular with maximum at 24 h as the length of the day. (D–E) Graph shows the phases of liver rhythmic proteins (blue) and their corresponding mRNAs (red) of secreted proteins (D) (11 in 201 cycling versus 29 in 3131 total dataset) as well as chaperones (C) (8 in 201 cycling versus 75 in 3131 total dataset). Lack of mRNA data indicates an arrhythmic transcript. Box plots at the bottom of each graph represent graphically the data of the mRNA (red) and protein (blue) phases. (TIF) [file pgen.1004047.s003.tif]

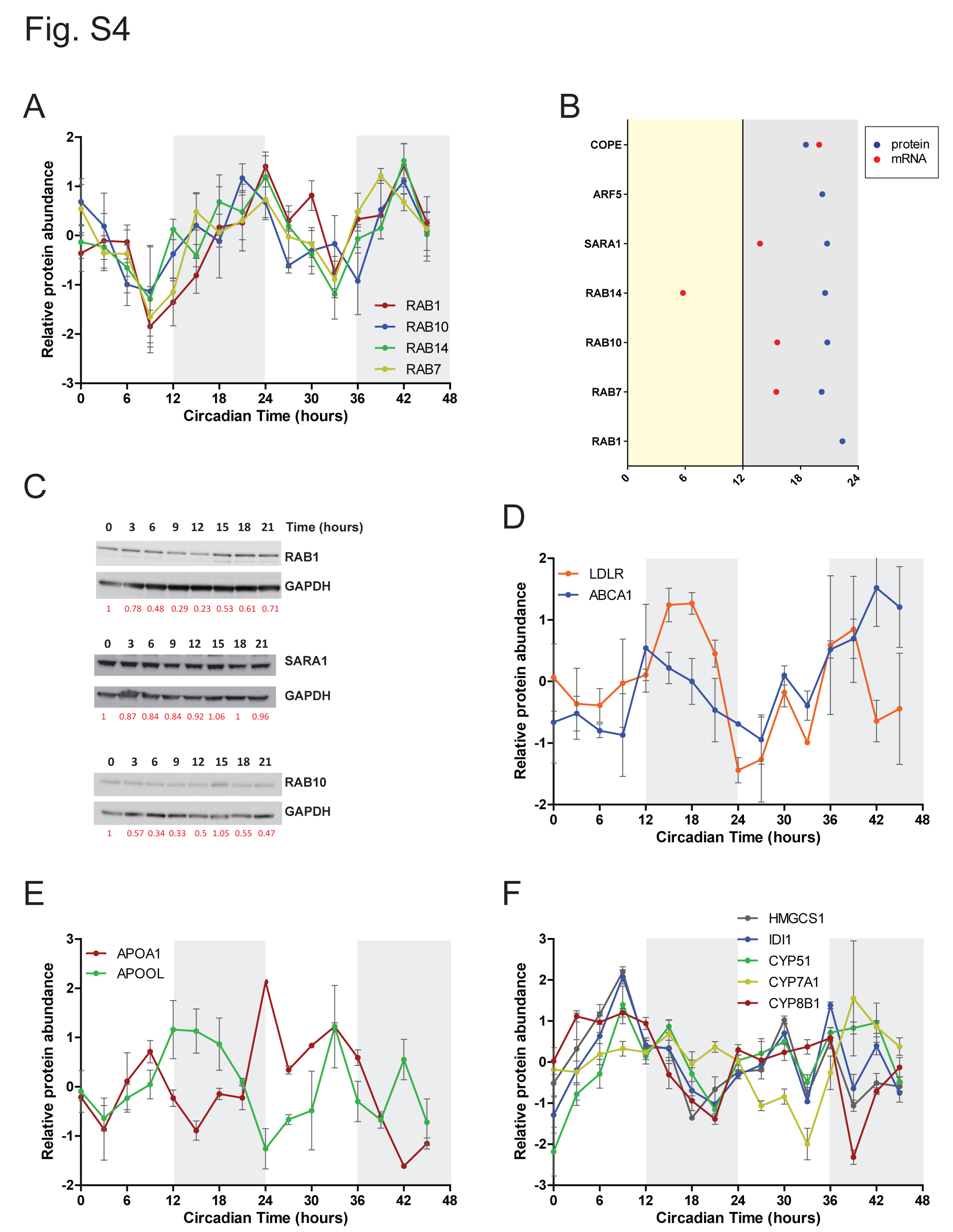

Supplement: Figure S4 — Coordinated abundance cycles of proteins involved in essential cellular processes. (A) Profiles of protein abundance, across the two sampled cycles, of cycling RAB GTPases in the mouse liver. Represented values correspond to the median of the normalized log2 z-scored ratios for each triplicate and their respective SEM. (B) Phases of cycling proteins (blue) and its correspondent mRNA (red) components of vesicle trafficking. Lack of mRNA data indicates arrhythmic transcript. (C) Western blots detecting RAB1, SARA1 and RAB10 in the samples of the first collected day. Loading control is shown with anti-GAPDH antibody. Numbers at the bottom indicate the relative density of the signal for each specific antibody at each time point normalized by GAPDH signal. (D–F) Profiles of abundance in the mouse liver for cycling proteins essential for cholesterol metabolism: hepatic receptors (D), apolipoproteins (E) and metabolic enzymes (F). Represented values correspond to the median of the normalized log2 z-scored ratios for each triplicate and their respective SEM. (TIF) [file pgen.1004047.s004.tif]

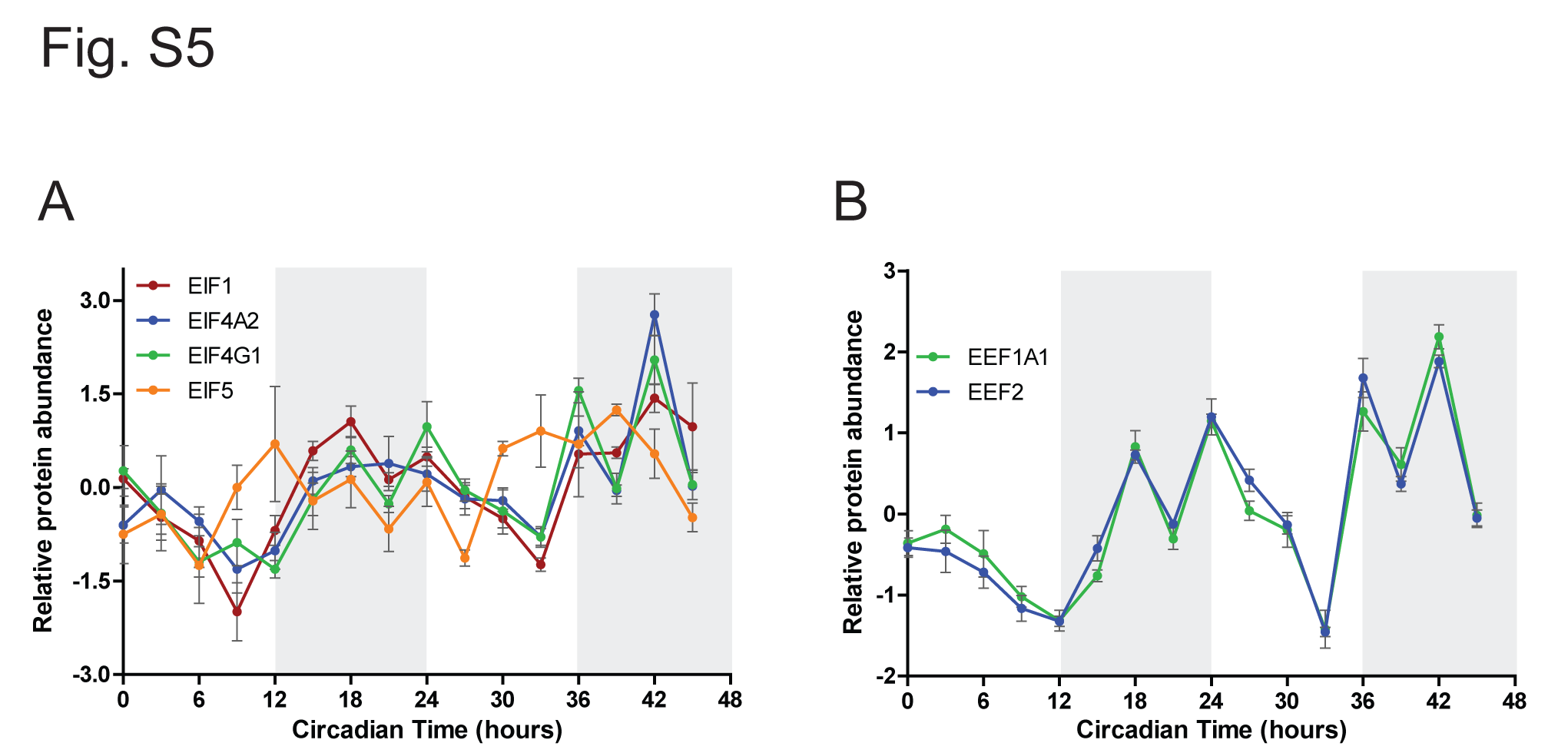

Supplement: Figure S5 — Circadian oscillations of abundances for crucial translation factors in the mouse liver. (A–B) Profiles of protein abundance across the two sampled cycles for essential initiation (A) and elongation (B) translation factors. (TIF) [file pgen.1004047.s005.tif]
